# Urease Expression in Pathogenic *Yersinia enterocolitica* Strains of Bio-Serotypes 2/O:9 and 1B/O:8 Is Differentially Regulated by the OmpR Regulator

**DOI:** 10.3389/fmicb.2020.00607

**Published:** 2020-04-08

**Authors:** Marta Nieckarz, Patrycja Kaczor, Karolina Jaworska, Adrianna Raczkowska, Katarzyna Brzostek

**Affiliations:** Department of Molecular Microbiology, Institute of Microbiology, Faculty of Biology, University of Warsaw, Warsaw, Poland

**Keywords:** *Yersinia enterocolitica*, OmpR regulator, urease activity, urease-subunits genes, proteome, strain-specific differences

## Abstract

*Yersinia enterocolitica* exhibits a dual lifestyle, existing as both a saprophyte and a pathogen colonizing different niches within a host organism. OmpR has been recognized as a regulator that controls the expression of genes involved in many different cellular processes and the virulence of pathogenic bacteria. Here, we have examined the influence of OmpR and varying temperature (26°C vs. 37°C) on the cytoplasmic proteome of *Y*. *enterocolitica* Ye9N (bio-serotype 2/O:9, low pathogenicity). Differential label-free quantitative proteomic analysis indicated that OmpR affects the cellular abundance of a number of proteins including subunits of urease, an enzyme that plays a significant role in acid tolerance and the pathogenicity of *Y. enterocolitica*. The impact of OmpR on the expression of urease under different growth conditions was studied in more detail by comparing urease activity and the transcription of *ure* genes in *Y. enterocolitica* strains Ye9N and Ye8N (highly pathogenic bio-serotype 1B/O:8). Urease expression was higher in strain Ye9N than in Ye8N and in cells grown at 26°C compared to 37°C. However, low pH, high osmolarity and the presence of urea did not have a clear effect on urease expression in either strain. Further analysis showed that OmpR participates in the positive regulation of three transcriptional units encoding the multi-subunit urease (*ureABC*, *ureEF*, and *ureGD*) in strain Ye9N, but this was not the case in strain Ye8N. Binding of OmpR to the *ureABC* and *ureEF* promoter regions was confirmed using an electrophoretic mobility shift assay, suggesting that this factor plays a direct role in regulating the transcription of these operons. In addition, we determined that OmpR modulates the expression of a *ureR*-like gene encoding a putative regulator of the *ure* gene cluster, but in the opposite manner, i.e., positively in Ye9N and negatively in Ye8N. These findings provide some novel insights into the function of OmpR in adaptation strategies of *Y. enterocolitica*.

## Introduction

*Yersinia enterocolitica*, a Gram-negative bacterium, exhibits a dual lifestyle, existing as both a saprophyte widely distributed in nature and a pathogen colonizing many different niches within host organisms. *Y. enterocolitica* has been isolated from different sources, including food, clinical material and certain warm- and cold-blooded animals (Fuchs et al., 2011; Fàbrega and Vila, 2012). In humans, *Y. enterocolitica* causes yersiniosis – an acute or chronic foodborne disease manifested by a variety of clinical symptoms, especially gastrointestinal (Bottone, 1997; Carniel et al., 2006). Cases of *y*ersiniosis occur worldwide, and in Europe it is the third most common food-borne gastroenteritis after campylobacteriosis and salmonellosis (European Food Safety Authority and European Centre for Disease Prevention, and Control [ECDC], 2017; Chlebicz and Śliżewska, 2018).

*Yersinia enterocolitica* is a member of the genus *Yersinia* (family Yersiniaceae, previously Enterobacteriaceae; Adeolu et al., 2016), which includes two other human pathogens: *Y. pseudotuberculosis* and the plague bacillus *Y. pestis*, and at least 18 other species considered harmless for humans (Bottone, 2015). *Y. enterocolitica* is a heterogeneous species comprising many bio-serotypes differing in pathogenicity (Howard et al., 2006). Of six biotypes (1A, 1B, 2, 3, 4 and 5) separated according to their biochemical properties, biotype 1A is considered non-pathogenic, biotype 1B is highly pathogenic to humans and lethal in a mouse model of infection, while biotypes 2 to 5 exhibit low virulence (unable to kill mice) (Bottone, 1997). Strains of biotypes 1A and 2−5 belong to the *Y. enterocolitica* subsp. *palearctica* (Neubauer et al., 2000) and are generally found in Europe and Japan (termed “Old World strains”). The most virulent biotype 1B strains belong to the *Y. enterocolitica* subsp. *enterocolitica* (Neubauer et al., 2000) and are dominant in North America (“New World strains”). *Y. enterocolitica* can be divided into approximately 70 serotypes, of which only eleven have been associated with disease in humans, with the majority of cases involving just four virulent serotypes: O:8 (biotype 1B), O:3 (biotype 4), O:9 (biotype 2), and O:5,27 (biotypes 2 and 3) (Bottone, 1997; Fàbrega and Vila, 2012).

*Yersinia enterocolitica* synthesizes numerous virulence factors that are active at different stages of an infection and whose expression is altered according to the diverse growth conditions experienced by these bacteria in mammalian hosts (Straley and Perry, 1995; Marceau, 2005; Erhardt and Dersch, 2015; Chen et al., 2016). It was previously demonstrated that a functional urease is essential for the survival of *Y. enterocolitica* O:9 during passage through the stomach (De Koning-Ward and Robins-Browne, 1995; Young et al., 1996), and is also necessary for full virulence in oral infections in the rat model (Gripenberg-Lerche et al., 2000). In addition, a non-enzymatic biological effect of urease may be to trigger the inflammatory process that is thought to underly the arthritogenicity of *Y. enterocolitica* (Probst et al., 1993). Ureases appear to play a vital role in the survival of *Y. enterocolitica* cells in the natural environment by degrading urea in the soil and water, which is utilized by this saprophyte as the sole nitrogen source (Young et al., 1996).

Ureases are highly conserved enzymes widely distributed among bacterial species (Mobley et al., 1995; Konieczna et al., 2012; Kappaun et al., 2018). They play an important role in acid resistance and the virulence of several human pathogens, including *Proteus mirabilis*, *Helicobacter pylori* and *Staphylococcus aureus* (Stingl et al., 2002; Rutherford, 2014; Zhou et al., 2019). Bacterial ureases are multi-subunit nickel-dependent enzymes which catalyze the hydrolysis of urea into ammonia (urea amidohydrolase, EC 3.5.1.5). They are typically comprised of three distinct subunits (UreA, subunit γ; UreB, subunit β; and UreC, subunit α) encoded by genes organized into an operon (Mobley et al., 1995; Konieczna et al., 2012). For assembly of the holoenzyme, four accessory/regulatory proteins are required, which are encoded by genes located in the same *ure* locus (Farrugia et al., 2013; Kappaun et al., 2018).

Different environmental conditions influence the production/activity of the urease complex in bacteria. Some bacterial ureases are regulated by nutrient conditions, pH, urea concentration or nitrogen limitation, whereas others are unaffected by environmental signals and are synthesized constitutively (Mobley et al., 1995; Patra and Aschenbach, 2018). Several regulators of *ure* gene expression have been identified in ureolytic bacteria. In *Klebsiella* species, the regulator NtrC activates transcription of the *ure* promoters in response to nitrogen deprivation (Collins et al., 1993; Magasanik, 1993). Urease expression in *Y. pseudotuberculosis* is positively regulated by the regulator OmpR and negatively by the regulators RovM and CsrA (Hu et al., 2009; Dai et al., 2018). In many ureolytic pathogenic bacteria, e.g., *P. mirabilis, Providencia stuartii, Escherichia coli* and *Salmonella* species, the regulator UreR positively influences expression of the *ure* genes in the presence of urea (Mobley et al., 1985; Mulrooney et al., 1988; D’Orazio and Collins, 1993; D’Orazio and Collins, 1995; Poore et al., 2001). UreR is a member of the AraC/XylS family of transcriptional regulators and is encoded by the *ureR* gene that can be located on the chromosome or a plasmid (Nicholson et al., 1993; Island and Mobley, 1995; Poore and Mobley, 2003).

The two-component regulatory system EnvZ/OmpR, consisting of the histidine kinase EnvZ and response regulator OmpR, is involved in the control of various cellular processes, and functions in a number of bacteria (Stock et al., 1989; Hoch and Silhavy, 1995). In response to changes in the environment (osmolarity and pH) the kinase EnvZ phosphorylates OmpR. Conformational changes in this phosphorylated regulator allow it to bind to DNA and modulate gene expression (Kenney, 2002). There is now a considerable body of evidence indicating that OmpR is involved in the regulation of fundamental physiological properties in *Yersinia* species (Gao et al., 2011; Gueguen et al., 2013; Reboul et al., 2014; Bontemps-Gallo et al., 2019). Our research on *Y. enterocolitica* strain Ye9N (bio-serotype 2/O:9) suggests that OmpR performs a variety of functions, some of which differ from those described in other bacteria (Brzostek et al., 2012). We have shown that OmpR plays a regulatory function in modulating the abundance of outer membrane proteins, including virulence factors like invasin Inv and adhesin YadA (Brzostek et al., 2007; Skorek et al., 2013; Nieckarz et al., 2016). In contrast to *E. coli*, we found that the OmpR of *Y. enterocolitica* is involved in the positive control of flagellum synthesis and motility (Raczkowska et al., 2011). OmpR-dependent regulation of the components of the *Y. enterocolitica* multidrug efflux pump AcrAB-TolC was also demonstrated (Raczkowska et al., 2015). Another study showed that an *ompR* mutant of *Y. enterocolitica* strain Ye9N exhibits a reduced ability to survive under acidic conditions *in vitro* and within macrophages, which indicates that OmpR is involved in the adaptation of this bacterium to low pH (Brzostek et al., 2003).

In this study, the impact of OmpR and varying temperature (26°C vs. 37°C) on the cytoplasmic proteome of strain Ye9N (2/O:9) was investigated by shotgun label-free quantitative proteomic analysis (LC-MS/MS). Our results indicated that the loss of OmpR in this strain affects the production of a number of proteins, including subunits of urease. In spite of significant advances in our understanding of urease function in *Y. enterocolitica*, the molecular mechanisms controlling *ure* expression remain unclear. Therefore, to verify the proteomic data and to gain a greater understanding of the role of OmpR in regulating urease production in *Y. enterocolitica*, urease activity and *ure* gene transcription were further examined in strain Ye9N (low pathogenic bio-serotype 2/O:9) and strain Ye8N (highly pathogenic bio-serotype 1B/O:8). Our investigations revealed that the regulator OmpR plays a somewhat different role in controlling urease expression in these two pathogenic *Y. enterocolitica* strains. These findings provide some novel insights into the function of OmpR in the adaptation strategy of *Y. enterocolitica*.

## Materials and Methods

### Bacterial Strains and Growth Conditions

The strains and plasmids used in this study are described in Supplementary Table S1. *Y. enterocolitica* strains were cultured at 26°C or 37°C in LB medium (10 g/l tryptone, 5 g/l yeast extract, 5 g/l NaCl). *E. coli* strains were grown at 37°C in LB medium. Antibiotics were used at the following concentrations: nalidixic acid (Nal) – 30 μg/ml, chloramphenicol (Cm) – 25 μg/ml, kanamycin (Km) – 50 μg/ml, gentamicin (Gm) – 40 μg/ml, tetracycline (Tet) – 12.5 μg/ml, trimethoprim (Tp) – 50 μg/ml.

### Isolation of Cytoplasmic Proteins for Shotgun Label-Free Quantitative Proteomic Analysis

Triplicate overnight cultures of *Y. enterocolitica* strains Ye9N (the wild-type strain) and AR4 (OmpR-deficient mutant) were grown in LB, pH 7.0 at 26°C or 37°C with shaking (150 rpm) to an OD_600_ of 3.0. Samples of 25 ml were centrifuged (8000 × *g*, 20 min, 4°C), and the cell pellets were flash frozen in liquid nitrogen and stored at −80°C prior to fractionation. Each of the bacterial pellets was resuspended in 12.5 ml of lysis buffer (200 mM Tris−HCl pH 8.0, 0.5 M sucrose, 250 μg/ml lysozyme, 1 mM EDTA, 1 mM phenylmethylsulfonyl fluoride) and incubated at 4°C for 1 h. The cell suspensions were then sonicated on ice for 18 cycles of 30 s, separated by 30 s rest intervals, using a Sonics Vibra-Cell VCX 130 (Sonics & Materials, Newtown, CT, United States). The cell lysates were centrifuged for 10 min at 8000 × *g* at 4°C to remove unbroken cells and debris, then the supernatants were centrifuged at high speed (35,000 × *g*, 1.5 h, 4°C) to pellet total membranes. The protein concentrations in the supernatants (cytoplasmic protein extracts) were measured using the RC-DC protein assay (Bio-Rad, Hercules, United States) and equalized by dilution with lysis buffer. The protein extracts were precipitated by adding 4 volumes of cold acetone and holding at −20°C overnight. The samples were then centrifuged for 20 min at 14,000 × *g* at 4°C. The resultant protein pellets were rinsed with cold acetone twice, each time resuspending and centrifuging for 20 min at 14,000 × *g* at 4°C. After carefully removing the supernatants, the protein precipitates were briefly air-dried and resuspended in 40 μl of 5× Invitrosol LC/MS Protein Solubilizer (Invitrogen Life Science Technologies). To each sample, 4 μl of 20% (w/v) urea stock solution was added to facilitate dissolution of the pellet. The samples were then vortexed for 3 min, incubated at 60°C for 25 min and diluted with 160 μl of 25 mM ammonium bicarbonate to produce a final Invitrosol concentration of 1×.

Sample preparation for mass spectrometry analyses were performed as described previously (Nieckarz et al., 2016). The cytoplasmic proteomes of strains Ye9N and AR4 were compared to produce a differential proteome list. Proteins whose abundance differed significantly (*q*-value ≤ 0.05) between the wild-type strain Ye9N and OmpR-deficient mutant AR4, according to MS analysis, were defined by the ratios of ≤0.67 (protein more abundant in AR4) or ≥1.5 (protein less abundant in AR4).

### Acid-Sensitivity Assay

The acid-sensitivity assay was performed essentially as described by Kakoschke et al. (2014). To measure survival in acid, overnight cultures grown in LB at 26°C or 37°C were diluted to 10^7^ CFU/ml in PBS (pH 7.0). Then, 0.5 ml of each bacterial cell suspension was mixed with an equal volume of PBS acidified with acetic acid to pH 4.0 (acid stress), or PBS at pH 7.0 (control). The cell suspensions were incubated at 26°C or 37°C for 90 min and then dilutions were plated on LB plates to determine the number of viable cells as CFU/ml. The percentage survival value was defined as the CFU/ml after acid treatment × 100/CFU/ml of the control.

### Molecular Biology Techniques

DNA manipulations, polymerase chain reactions (PCRs), restriction digests, ligations and DNA electrophoresis, were performed according to standard protocols (Sambrook and Russell, 2001). Plasmid and genomic DNA were isolated using a Plasmid Miniprep DNA purification Kit and GeneMATRIX Bacterial & Yeast Genomic DNA Purification Kit (EurX), respectively. When amplified fragments were used for cloning, the PCR was performed using DreamTaq DNA polymerase or Phusion High-Fidelity DNA polymerase (Thermo Scientific). Oligonucleotide primers for PCR and sequencing were purchased from Sigma Aldrich and are listed in Supplementary Table S2. DNA fragments amplified by PCR were purified using a PCR/DNA Clean Up kit (EurX). The plasmids used in this study are described in Supplementary Table S1. DNA sequencing was performed by Genomed S.A. (Warsaw, Poland).

### Construction of *ureABC*:*lacZ*, *ureEF:lacZ*, *ureGD:lacZ*, and *ureR:lacZ* Transcriptional Fusion Plasmids

To obtain *ureABC*:*lacZ*, *ureEF:lacZ*, *ureGD:lacZ* and *ureR:lacZ* transcriptional fusions, DNA fragments containing the promoters of the *ureABC*, *ureEF* and *ureGD* operons, and the *ureR*-like gene were amplified from *Y. enterocolitica* chromosomal DNA by PCR using the primer pairs NureABCKpnIL/NureABCKpnIP, LureEFEcoRI/PureEFKpnI, LureGDEcoRI/PureGDKpnI, and EcoRIReg1/KpnIReg2, respectively (Supplementary Table S2). The amplified fragments were digested with *Kpn*I (in the case of the *ureABC* operon promoter) or *Eco*RI/*Kpn*I and cloned into the corresponding sites of reporter vector pCM132Gm [derivative of plasmid pCM132 (Marx and Lidstrom, 2001) containing a gentamicin resistance cassette], upstream of a promoterless *lacZ* gene. The resulting constructs were verified by PCR using the primer pair pCM132GmSPR1/pCM132GmSPR2 (flanking the *Eco*RI and *Kpn*I recognition sequences) followed by sequencing of the amplicons. The correct orientation of the cloned *ureABC* promoter was verified by PCR using the primer pair pCM132GmSPR1/lacZSprP and by sequencing. The constructs pCM132Gm-*ureABC:lacZ*, pCM132Gm-*ureEF:lacZ*, pCM132Gm-*ureGD:lacZ* and pCM132Gm-*ureR:lacZ* were introduced into *E. coli* CC118 λ*pir* and transferred by conjugation into *Y. enterocolitica* Ye9N, the *ompR* mutant AR4, Ye8N and the *ompB* mutant KJ4, selecting transconjugants on LB plates containing Gm and Nal. The presence of these constructs in the *Y. enterocolitica* strains was confirmed by plasmid isolation and PCR with the primer pair pCM132GmSPR1/pCM132GmSPR2.

### β-Galactosidase Assays

β-galactosidase assays were performed essentially as described by Thibodeau et al. (2004), using 96-well flat-bottomed plates (Nest Sc. Biotech.) and a Sunrise plate reader (Tecan). Briefly, cultures grown to stationary phase at 26°C or 37°C were diluted with LB medium to an OD_600_ of 0.4–0.5 and 80 μl of each cell suspension were then mixed with 20 μl of POPCulture Reagent (EMD Millipore Corp) and incubated for 15 min to cause cell lysis. In the wells of a microtiter plate, 20 μl of each cell lysate were mixed with 130 μl of Z Buffer and 30 μl of ONPG (4 mg/ml). For kinetic assays, the absorbance at 415 nm (relative to a blank) was measured at time intervals of 10 s, with 2 s of shaking before each reading. The assays were performed at 26°C and monitored for up to 20 min. The β-galactosidase activity was expressed in Miller units calculated as described previously (Thibodeau et al., 2004). Each assay was performed at least in triplicate. To test the effect of acid pH, high osmolarity and urea on reporter gene expression, cultures grown to stationary phase at 26°C were diluted to an OD_600_ of ∼ 0.4 in LB medium. Then 1 ml of each culture was centrifuged (4000 × *g*, 5 min, room temperature) and the cell pellets were resuspended in 1 ml of LB (86 mM NaCl, pH 7.0), LB adjusted to pH 4.5, LB supplemented with NaCl to a final concentration of 350 mM or LB containing 100 mM urea. These cultures were incubated for 2 h at 26°C with shaking (150 rpm). After two washes in LB medium by centrifugation (4000 × *g*, 5 min, room temperature) and gentle re-suspension, β-galactosidase activity in the final cell suspensions was measured.

### Electrophoretic Mobility Shift Assays (EMSAs)

The *in vitro* interaction between phosphorylated OmpR (OmpR-P) and the promoters of selected genes was examined using EMSAs, as described previously (Jaworska et al., 2018), with some modifications. The primers listed in Supplementary Table S2 were used in PCRs with *Y. enterocolitica* Ye9N genomic DNA to amplify DNA fragments comprising the regulatory regions of the operons *ureABC* (−305 to + 7 relative to the A of the ATG start codon of *ureA*), *ureEF* (−352 to + 33 relative to the A of the ATG start codon of *ureE*), *ureGD* (−272 to + 33 relative to the A of the ATG start codon of *ureG*), and gene *ureR*-like (−310 to + 52 relative to the A of the ATG start codon of *ureR*-like). Increasing amounts of recombinant OmpR-His_6_ were phosphorylated *in vitro* by incubation for 30 min at room temperature in reaction buffer (50 mM Tris-HCl pH 8.0, 100 mM KCl, 1 mM EDTA, 1 mM DTT, 20 mM MgCl_2_, 12% glycerol, 100 μg/ml BSA, 0.1% Triton X-100) containing 20 mM acetyl phosphate (lithium potassium acetyl phosphate; Sigma-Aldrich). After phosphorylation, 0.05 pmoles of the respective test fragments were added to form the separate EMSA binding reactions. As a negative control, a 211-bp fragment of the *Y. enterocolitica* 16S rRNA gene amplified by PCR (Supplementary Table S2) was also included in each reaction. The binding reactions were analyzed by electrophoresis (∼3 h at 110V) on 4.2% native polyacrylamide gels (19:1 acrylamide/bis-acrylamide, 0.2 × TBE, 2% glycerol). SYBRgreen 1× solution (Invitrogen) was used to stain the DNA bands in the gels, which were visualized with a GE Healthcare AI600 imager.

### Semi-Quantitative Urease Enzyme Assay

Overnight cultures of *Y. enterocolitica* strains grown in LB, pH 7.0 at 26°C or 37°C were adjusted to an OD_600_ of 1.0 (approximately 10^9^ bacterial cells per ml). Then 1 ml of each cell suspension was centrifuged (4000 × *g*, 3 min, 25°C). The cell pellets were resuspended in 0.1 ml of LB and 10 μl lots were spotted onto the surface of urease enzyme assay plates [Christensen’s urea agar – 1 g/L peptone, 1 g/L D(+)-glucose, 2 g/L potassium dihydrogen phosphate, 5 g/L sodium chloride, 0.012 g/L phenol red, 12 g/L agar, pH 6.8 with or without 2% urea]. Following incubation at 26°C or 37°C for 24 h, urease activity was observed as pink zones around the bacterial growth. Urease catalyzes the hydrolysis of urea to ammonia, which causes an increase in pH indicated by the pH indicator phenol red (Young et al., 1996).

### Quantitative Urease Enzyme Assay

The quantitative urease activity assay was based on the measurement of ammonia formed by enzymatic hydrolysis of urea, using Nessler’s reagent. Briefly, triplicate cultures of the *Y. enterocolitica* strains were grown overnight at 26°C in LB medium with shaking (150 rpm). Then, 1 ml of each culture was centrifuged (4000 × *g*, 3 min, 25°C) and the cell pellets washed once in Christensen’s urea medium [1 g/L peptone, 1 g/L D(+)-glucose, 2 g/L potassium dihydrogen phosphate, 5 g/L sodium chloride pH 6.8, with 2% urea] and resuspended in the same. The cell suspensions were diluted to an OD_600_ of 0.1 in Christensen’s urea medium and incubated for 2 h at 26°C with shaking (150 rpm). The OD_600_ of the cultures was measured to confirm no change, then 90 μl aliquots were mixed with 180 μl of 10% Nessler’s reagent (Sigma Aldrich) in the wells of a 96-well flat-bottomed plate (Nest Sc. Biotech.). Controls containing only Christensen’s urea medium were also included. The absorbance at 420 nm (relative to a blank) was measured 5 min after mixing the cells with Nessler’s reagent, using a TECAN Infinite M200PRO microplate reader. The levels of ammonia produced were determined from a standard curve constructed using defined concentrations of NH_4_Cl prepared fresh in the assay buffer (Young et al., 1996).

### Construction of an *ompB* (*ompRenvZ*) Deletion Mutant

The *ompB*:Tp deletion mutant of *Y. enterocolitica* Ye8N (replacement with Tp resistance gene cassette) was obtained by homologous recombination using suicide vector pDS132 (Philippe et al., 2004), carrying the mutagenic DNA fragment constructed by overlap extension PCR using primers listed in Supplementary Table S2. Briefly, three DNA fragments were PCR-amplified using strain Ye8N genomic DNA (for flanking regions) or plasmid p34E-Tp (for the Tp cassette) as the templates. A mixture of these three amplicons was then used as the template with flanking primers in a PCR. The mutagenic fragment was cloned into pDS132. The resulting construct pDSompB was transferred from *E. coli* CC118 λ*pir* to *Y. enterocolitica* strain Ye8N by triparental mating, with *E. coli* strain DH5α carrying plasmid pRK2013 as a helper. To select for the second recombination, the transconjugant strains were plated on LB agar containing Tp and 10% (w/v) sucrose. Sucrose-resistant colonies were screened for the loss of the plasmid (Cm resistance). The correct allelic exchange was verified by PCR using primers listed in Supplementary Table S2 and by sequencing.

### Construction of Plasmid pompB for Complementation

To complement the *ompB* mutation, the coding sequence of the *ompB* (*ompRenvZ*) operon with the native ribosome binding site was amplified by PCR using Ye8N chromosomal DNA as the template with primers OmpB1 and OmpB2 (Supplementary Table S2). The amplified fragment was digested with *Eco*RI/*Bam*HI and cloned into the corresponding sites of reporter vector pBBR1MCS-2, containing a kanamycin resistance cassette (Kovach et al., 1995). The resulting construct pompB was verified by sequencing and used to transform *E. coli* S17 λ*pir*. This plasmid was then introduced into the *ompB* mutant strain KJ4 by biparental conjugation. Exconjugants were selected on LB agar plates containing trimethoprim and kanamycin.

### Bioinformatic Analyses

Bioinformatic analyses of the mass spectrometric data were performed as described previously (Nieckarz et al., 2016). *In silico* analyses of the *ure* gene cluster and *ureR*-like gene in *Y. enterocolitica* strains were performed on a shotgun genome sequence of *Y. enterocolitica* subsp. *palearctica* Ye9N (bio-serotype 2/O:9; NCBI/GenBank: JAALCX000000000) and *Y. enterocolitica* subsp. *enterocolitica* 8081 (bio-serotype 1B/O:8; NCBI/GenBank: AM286415). The Needleman-Wunsch algorithm was used for sequence alignment (Altschul et al., 1997). Promoter prediction was performed using the web-based software BPROM in the Softberry package (Solovyev and Salamov, 2011). Manual homology searches of predicted urease sub-units were conducted by BLAST analysis^1^ to describe a urease protein complex. The MOTIF bioinformatics tool provided by GenomeNet, Japan, was used to search for sequence motifs^2^. The identified proteins were described according to the UniProt databases or GenBank, or homologous sequences obtained using BLAST. Statistical analyses were performed using Prism 5 software (v. 5.01, GraphPad). Student’s *t*-test was used to determine statistically significant differences.

## Results

### Effect of Temperature on the Cytoplasmic Proteome of *Y. enterocolitica* Strain Ye9N

To study the effect of temperature on the cytoplasmic proteome of *Y. enterocolitica*, cells of wild-type strain Ye9N, grown in standard lysogeny broth (LB) medium at 26°C or 37°C, were fractionated using ultracentrifugation and the cytoplasmic fraction was analyzed by shotgun label-free quantitative LC-MS/MS. This analysis revealed 23 differentially expressed proteins (*q*-value ≤ 0.05, at least two peptides per protein, minimal acceptable fold change 1.5, Table 1). The more abundant proteins at the higher growth temperature included molecular chaperones such as GroEL (Hsp60; ∼4-fold) together with its co-chaperone GroES (∼7-fold), DnaK (Hsp70, ∼1.7-fold), ClpB (∼6-fold) and HtpG, a prokaryotic Hsp90 homolog (∼4-fold). In contrast, the level of trigger factor (TF), a ribosome-associated peptidyl *cis/trans* isomerase, which represents the only ribosome-associated chaperone known in bacteria (Hoffmann et al., 2010), was decreased at 37°C (∼2.7-fold). The level of YopE, the secreted effector of the *Yersinia* Ysc-Yop T3SS, which disrupts the actin cytoskeleton of eukaryotic cells (Viboud and Bliska, 2005; Aepfelbacher et al., 2007; Trosky et al., 2008), was higher at 37°C compared to 26°C (∼9-fold), confirming previous reports (Lambert de Rouvroit et al., 1992; Akopyan et al., 2011). Upregulation of YerA (∼6.7-fold), a chaperone required for YopE secretion at 37°C, was also noted (Parsot et al., 2003). Proteins more abundant at the lower temperature (26°C) included the periplasmic maltose-binding protein MalE (∼14.39-fold), amino acid metabolic enzymes histidine ammonia-lyase HutH (∼7.57-fold) and succinylornithine transaminase AstC (∼7.99-fold), as well as bacterioferritin Bfr (∼7.43-fold). Interestingly, this proteomic analysis revealed downregulation at 37°C of a structural subunit of urease, UreC (∼2.3-fold), and the urease accessory proteins UreE (∼2.8-fold) and UreG (∼3.2-fold).

**TABLE 1:** Comparison of the cytoplasmic proteins produced by *Y. enterocolitica* wild-type strain Ye9N grown in LB medium at 26°C and 37°C.

Differentially expressed proteins		Regulation Ye9N^b^ 26°C vs. 37°C			
Accession no.	Protein description^a^	*q*-value^c^	ratio^d^	fc^e^	pep^f^
ADZ40805	Acetyl-coenzyme A synthetase AcsA	0.00098	2.47	2.47	12
ADZ44135	Bacterioferritin Bfr	0.04926	7.43	7.43	4
ADZ41157	Chaperone protein ClpB	0.02673	0.16	6.32	15
ADZ41113	Chaperone protein DnaK	0.00106	0.60	1.68	22
ADZ40874	Chaperonin GroEL	0.00007	0.26	3.91	34
ADZ40873	Co-chaperonin GroES	0.03221	0.15	6.88	4
ADZ43135	Deoxyribose-phosphate aldolase DeoC	0.02704	0.26	3.88	10
ADZ41668	Heat shock protein 90 homolog HtpG	0.00007	0.27	3.76	30
ADZ44331	Histidine ammonia-lyase HutH	0.01002	7.57	7.57	16
ADZ42026	Hypothetical protein	0.01577	0.12	8.12	6
ADZ44078	Maltose-binding periplasmic protein MalE	0.00007	14.39	14.39	7
ADZ40957	Polynucleotide phosphorylase/polyadenylase	0.03548	1.82	1.82	18
ADZ41611	Putative nucleotide-binding protein	0.02977	1.90	1.90	12
ADZ41171	S-ribosylhomocysteine lyase	0.04216	2.32	2.32	11
ADZ42349	Succinylornithine transaminase AstC	0.00031	7.99	7.99	10
ADZ44359	Superoxide dismutase [Mn] SodA	0.03798	3.38	3.38	14
ADZ42544	Transcriptional regulator SlyA	0.03673	3.24	3.24	5
ADZ41622	Trigger factor TF	0.00007	2.73	2.73	40
ADZ44485	Type III secretion system effector protein YopE	0.00007	0.11	9.35	20
ADZ43622	**Urease accessory protein UreE**	0.00007	2.81	2.81	17
ADZ43620	**Urease accessory protein UreG**	0.00659	3.17	3.17	12
ADZ43623	**Urease subunit alpha UreC**	0.03164	2.27	2.27	19
ADZ44484	YopE regulator YerA	0.00109	0.15	6.65	9

*^a^Description of the identified proteins according to the UniProt databases, GenBank, or homologous sequences obtained using BLAST. “Urease proteins” are shown in bold. ^b^Proteins whose production differed according to growth temperature (26°C vs. 37°C). ^c^q-value ≤ 0.05, statistically significant differences in abundance. ^d^Significant changes in protein abundance are defined by a ratio of ≤0.67 (protein more abundant at 37°C) or ≥1.5 (protein less abundant at 37°C, shaded gray). ^e^fc, fold change ≥1.5. ^f^pep, number of identified peptides belonging to the differentially produced protein.*

### Differences in Cytoplasmic Protein Abundance Between the Parental *Y. enterocolitica* Strain and an *ompR* Mutant

Shotgun Proteomic analysis was applied to identify cytoplasmic proteins subject to regulation by OmpR. We analyzed cytoplasmic fractions of the wild-type strain Ye9N and the isogenic *ompR* null mutant AR4, cultured at 26°C or 37°C in LB medium, by LC-MS/MS analysis. The mutant AR4 (Δ*ompR*:Km) was constructed previously by a reverse genetics PCR-based strategy (Brzostek et al., 2003). The proteomic analysis revealed 28 differentially expressed proteins (*q*-value ≤ 0.05, at least two peptides per protein, minimal acceptable fold change 1.5, Table 2). The major difference in cytoplasmic protein abundance between the strains at 26°C, was a more than 130-fold decrease in the level of HU-alpha, a subunit of histone-like bacterial protein important in maintenance of the nucleoid structure (Boubrik et al., 1991; Zhou et al., 2005; Kamashev et al., 2017), in the *ompR* mutant. This indicated that OmpR positively affects *hupA* gene expression. It was previously demonstrated that the *Y. pestis* PhoP protein, which belongs to the OmpR-family of two-component system response regulators, positively influences the production of HU-alpha (Zhou et al., 2005). Differential quantitative LC-MS/MS analysis of cytoplasmic samples revealed that urease subunit gamma UreA was less abundant (∼3-fold) in the *ompR*-negative strain AR4 compared with the parental strain at 26°C, suggesting positive OmpR-dependent regulation.

**TABLE 2:** Comparison of the cytoplasmic proteins produced by *Y. enterocolitica* Ye9N and isogenic *ompR* mutant AR4 at 26°C and 37°C.

Differentially expressed proteins		Regulation Ye9N vs. AR4^b^			
Accession no.	Protein description^a^	*q*-value^c^	ratio^d^	fc^e^	pep^f^
**at 26°C**					
ADZ40799	DNA-binding protein HU-alpha	0.00025	132.63	132.63	5
ADZ41266	Tryptophanase TnaA	0.03760	2.80	2.80	12
ADZ43625	**Urease subunit gamma UreA**	0.00299	3.10	3.10	11
**at 37°C**					
ADZ43844	2,5-diketo-D-gluconate reductase A	0.03179	6.83	6.83	4
ADZ43080	30S ribosomal protein S1	0.03179	0.51	1.98	17
ADZ44113	30S ribosomal protein S13	0.02542	0.30	3.34	7
ADZ44139	30S ribosomal protein S7	0.01070	0.25	4.08	8
ADZ44120	30S ribosomal protein S8	0.04965	0.30	3.35	11
ADZ44109	50S ribosomal protein L17	0.03550	0.42	2.39	9
ADZ44128	50S ribosomal protein L22	0.02814	0.18	5.69	8
ADZ44125	50S ribosomal protein L29	0.04842	0.14	7.08	8
ADZ41157	Chaperone protein ClpB	0.02083	2.33	2.33	17
ADZ40874	Chaperonin GroEL	0.04972	0.08	12.63	44
ADZ41961	D-galactose-binding periplasmic protein MglB	0.01663	0.40	2.48	10
ADZ41366	Glycine cleavage system H protein GcvH	0.03208	5.50	5.50	3
ADZ41367	Glycine dehydrogenase GcvP	0.02024	11.75	11.75	16
ADZ41668	Heat shock protein 90 homolog HtpG	0.01949	0.62	1.60	16
ADZ42015	Histidinol dehydrogenase	0.02661	0.30	3.36	4
ADZ43969	2-iminobutanoate/2-iminopropanoate deaminase	0.02562	4.54	4.54	3
ADZ44196	Phosphoenolpyruvate carboxykinase [ATP] PckA	0.01090	2.98	2.98	29
ADZ43449	Phosphoribosylaminoimidazole-succinocarboxamide synthase PurC	0.04457	4.95	4.95	6
ADZ44444	Transmembrane effector protein YopB	0.01229	0.21	4.70	12
ADZ43962	Putative sigma(54) modulation protein	0.01958	13.60	13.60	2
ADZ41622	Trigger factor	0.01518	0.57	1.77	24
ADZ43459	Uracil phosphoribosyltransferase	0.03418	3.35	3.35	14
ADZ43623	**Urease subunit alpha UreC**	0.00012	6.16	6.16	19
ADZ44446	Virulence-associated V antigen LcrV	0.00982	0.17	5.78	17
ADZ44470	Yop proteins translocation protein F YscF	0.01124	0.02	45.41	9

*^a^Description of the identified proteins according to the UniProt databases, GenBank, or homologous sequences obtained using BLAST. “Urease proteins” are shown in bold. ^b^Proteins whose abundance differed between the wild-type strain Ye9N and OmpR-deficient mutant AR4, grown at 26°C and 37°C. ^c^q-value ≤ 0.05, statistically significant differences in abundance. ^d^Significant changes in protein abundance are defined by a ratio of ≤0.67 (protein more abundant in OmpR-deficient mutant AR4) or ≥1.5 (protein less abundant in OmpR-deficient mutant AR4, shaded gray). ^e^fc, fold change ≥1.5. ^f^pep, number of identified peptides belonging to the differentially produced protein.*

The proteomic analysis of cytoplasmic fractions from cells grown at 37°C suggested that OmpR exerts a positive influence on the expression of the structural subunit of urease, UreC, since levels of this protein were reduced in the *ompR* mutant at this temperature (∼6-fold). In addition, we observed a two-fold decrease in the abundance of the chaperone ClpB in the *ompR* mutant. Proteins displaying reduced levels in the *ompR* mutant at 37°C included the putative sigma(54) modulation protein (∼13.6-fold) and the housekeeping enzymes 2,5-diketo-D-gluconate reductase A (∼6.8-fold) and glycine dehydrogenase GcvP (∼11.8-fold). In agreement with our previous finding, the absence of the OmpR regulator also caused an increase in the proteins of the Ysc-Yop T3SS (Nieckarz et al., 2016). These subunits of the *Yersinia* injectisome, which translocates the effector Yop proteins into host cells, include the translocation pore protein YopB (∼4.7-fold), forming a pore in the host cell membrane, the needle tip protein LcrV (∼5.8-fold) and the protein YscF, which polymerizes into a needle-like structure (45-fold) (Lee et al., 2000; Nordfelth and Wolf-Watz, 2001; Dewoody et al., 2013). Our proteomic data also revealed an increased amount of ribosomal proteins in the *ompR* mutant strain AR4 compared to the parent strain Ye9N at 37°C (∼ 2- to 7-fold), as well as higher levels of the chaperones GroEL (∼12.6-fold) and HtpG (∼1.6-fold).

The results of the proteomic analysis indicated that OmpR affects (both positively and negatively) the production of a number of proteins that serve a variety of functions. Notably, two of the three structural subunits of urease (UreA and UreC) were identified among the positively regulated OmpR-dependent targets.

### Temperature and OmpR Influence the Production of Urease in *Y. enterocolitica*

To investigate the influence of OmpR on the production of urease by *Y. enterocolitica* we examined the activity of this enzyme in the wild-type strains Ye9N (a low-level pathogenic strain of bio-serotype 2/O:9) and Ye8N (a high-level pathogenic strain of bio-serotype 1B/O:8), and their respective mutants AR4 (Δ*ompR*:Km) and KJ4. The mutant KJ4, lacking the *ompB* operon (Δ*ompRenvZ*:Tp), was constructed in the present study by targeted deletion via homologous recombination (see MATERIALS AND METHODS).

**FIGURE 1:**
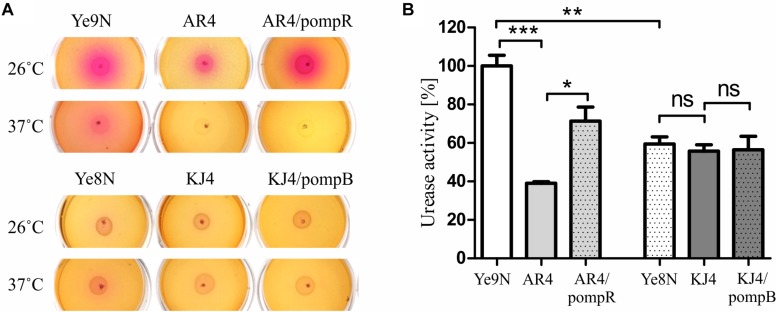
Changes in urease activity in *Y. enterocolitica* strains differing in their OmpR content. Ureolytic activity was compared in the strains Ye9N (wt), AR4 (*ompR*), complemented AR4 (*ompR*/pompR), Ye8N (wt), KJ4 (*ompB*), complemented KJ4 (*ompB*/pompB) **(A)** Results of a semi-quantitative urease activity assay based on a color change in Christensen’s urea agar after 24-h growth at 26°C or 37°C. **(B)** Quantitative urease activity determined using an assay based on the measurement of ammonia formed by enzymatic hydrolysis of urea in cultures grown at 26°C in Christensen’s medium supplemented with 2% of urea, using Nessler’s reagent. The concentration of ammonium ions is presented as a percentage, taking the value for the wild-type strain Ye9N as 100%. Significance was calculated using Student’s unpaired *t*-test (***P ≤ 0.001; **P ≤ 0.01; and *P ≤ 0.05; ns, not significant, *P* > 0.05). Data are from three independent experiments.

Ureolytic activity was estimated in a semi-quantitative manner by plating *Y. enterocolitica* strains on Christensen’s urea agar and monitoring the change in color after 24-h incubation at 26°C or 37°C (Figure 1A). Higher urease activity was observed in strain Ye9N than in Ye8N at both temperatures. Moreover, the ureolytic activity of Ye9N was higher at 26°C than 37°C. The *ompR* mutant (AR4) exhibited a decreased level of urease production compared to Ye9N at both temperatures. In contrast, no visible difference was observed between Ye8N and KJ4 (*ompB*), irrespective of the growth temperature. To confirm that OmpR promotes urease production in Ye9N, plasmid pompR carrying the wild-type *ompR* allele was used to complement the mutation in strain AR4. Complementation caused a significant increase in urease activity in this *ompR* mutant at 26°C but not at 37°C. However, expression of the *ompB* operon *in trans* (plasmid pompB carrying both the *ompR* and *envZ* genes) in the *ompB* mutant KJ4, did not affect urease activity at either of the tested temperatures.

To further examine the impact of OmpR on the production of urease we employed a quantitative assay using Nessler’s reagent. The results presented in Figure 1B confirmed the Christensen’s urea agar data. Strain Ye9N displayed the highest urease activity, and that detected in the *ompR*-deficient strain AR4 was significantly lower (2.6-fold). Moreover, complementation of the *ompR* mutation in strain AR4 caused a clearly visible increase in urease activity (1.8-fold). There was no difference in urease activity between strains Ye8N, mutant KJ4 and KJ4/pompB. These findings showed that the level of ureolytic activity is dependent on the *Y. enterocolitica* strain, the presence of OmpR protein, as well as the temperature.

### OmpR Enhances the Survival of *Y. enterocolitica* Strains Ye9N and Ye8N at Acidic pH, but to Different Degrees

**FIGURE 2:**
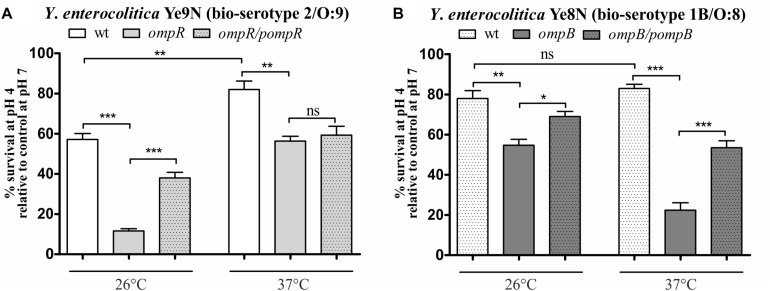
OmpR-dependent survival of *Y. enterocolitica* strains of bio-serotypes 2/O:9 **(A)** and 1B/O:8 **(B)** upon exposure to acid stress at 26°C or 37°C. The low pH sensitivity of the following strains was compared: Ye9N (2/O:9, wt), AR4 (*ompR* mutant), complemented AR4 (*ompR*/pompR), Ye8N (1B/O:8, wt), KJ4 (*ompB* mutant), complemented KJ4 (*ompB*/pompB). Bacterial viability following 90-min exposure to pH 4.0 is expressed relative to that of cells incubated in PBS at pH 7.0 (control). The data represent mean values with standard deviations, obtained in at least three independent experiments. Significance was calculated using Student’s unpaired *t*-test (***P ≤ 0.001; **P ≤ 0.01; and *P ≤ 0.05; ns, not significant, *P* > 0.05).

To examine whether there are other phenotypic differences between the *Y. enterocolitica* strains Ye9N and Ye8N, their survival abilities at acidic pH was analyzed. It was previously demonstrated that the regulator OmpR is necessary for the survival of *Y. enterocolitica* strains of bio-serotypes 1B/O:8 and 2/O:9 in acidic conditions *in vitro* (Dorrell et al., 1998; Brzostek et al., 2003). However, in these separate studies, the acid stress conditions and temperatures applied to these strains were different. Thus, it was thought necessary to examine the effect of OmpR on the susceptibility to acid stress of strains Ye9N and Ye8N by studying them in parallel. The acid survival abilities of these strains and their OmpR-deficient mutants were analyzed by exposing cells grown at 26°C or 37°C to pH 4.0 for 90 min, and then determining the viable cell number. This was expressed as a percentage of the number of viable cells following exposure to pH 7.0 for the same length of time (Figure 2). Compared to wild-type strain Ye9N, its *ompR* mutant AR4 displayed decreases in% survival in acid conditions of 46% at 26°C and 26% at 37°C (Figure 2A). Similarly, the OmpR-deficient mutant KJ4, exhibited decreased survival in acid conditions compared to wild-type parent strain Ye8N: by 23% at 26°C and 61% at 37°C (Figure 2B). To confirm that OmpR promotes acid survival in *Y*. *enterocolitica*, plasmids pompR and pompB carrying the wild-type *ompR* alleles were used to complement the mutations in strain AR4 and KJ4, respectively. Complementation caused a significant increase in survival at pH 4.0, except for strain AR4 grown at 37°C. Together, these findings showed that both *Y. enterocolitica* strains are able to survive acid shock at the two tested temperatures, but strain Ye9N is more acid sensitive than Ye8N at 26°C. Moreover, OmpR promotes resistance to acidic stress. Strong OmpR-dependent acid survival was observed for strain Ye9N at 26°C and for strain Ye8N at 37°C. Thus, the impact of this regulator on acid survival is both strain specific and affected by temperature.

### Genomic Organization of the *ure* Locus of *Y. enterocolitica* and Sequence Comparisons

**FIGURE 3:**
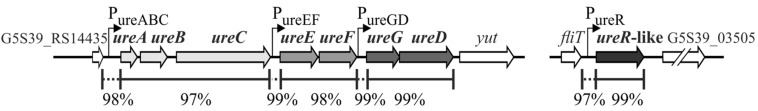
The genomic organization of the *ure* gene cluster and *ureR*-like gene in *Y. enterocolitica* strain Ye9N (2/O:9) (NCBI/GenBank: JAALCX010000039 and NCBI/GenBank: JAALCX010000005, respectively). Percentage identity of the DNA sequences of the Ye9N strain with the equivalent sequences of Ye8N (1B/O:8) (derivative of 8081, NCBI/GenBank: AM286415.1) is shown. Intergenic regions between the operons/genes are marked by dotted lines. The *ure* gene cluster is flanked upstream by the gene encoding a hypothetical protein (G5S39_RS14435) and downstream by the *yut* gene. The *ureR*-like gene is localized between the *fliT* gene and the gene encoding a metal-dependent phosphohydrolase (G5S39_03505). The positions of putative promoters are indicated by arrows. The locus tags in the Ye9N strain are indicated.

A shotgun genome sequence has recently been generated for *Y. enterocolitica* strain Ye9N (2/O:9). The availability of raw sequence data files of the Ye9N genome (NCBI/GenBank: JAALCX000000000) allowed us to examine the *ure* locus (contig 39) and compare it to that of strain 8081 (1B/O:8, NCBI/GenBank: AM286415,Thomson et al., 2006), whose derivative is strain Ye8N. Bioinformatic analysis revealed the presence of chromosomally located multigenic *ure* loci in both strains (Figure 3 and Supplementary Figure S1A). Within each locus, the *ure* genes are all in the same transcriptional orientation. In these strains, the *ure* locus is flanked by conserved genes: upstream is a gene encoding a hypothetical protein, while downstream there is the *yut* gene encoding a urea transporter *(*Sebbane et al., 2002). Using BPROM software, three putative promoters (−10 and −35 motifs) located upstream of *ureA*, *ureE* and *ureG* were identified within the urease cluster of both strains (Figure 3 and Supplementary Figure S1A), suggesting its organization in three operons, *ureABC*, *ureEF* and *ureGD*, encoding the structural (UreABC) and accessory/regulatory proteins (UreEF and UreGD). Alignment of the nucleotide sequences of *ureABC*, *ureEF* and *ureGD* of strain Ye9N with whose of strain 8081 showed 97%, 98% and 99% sequence identity, respectively. Nucleotide sequence alignments of the intergenic region upstream of *ureABC* of the analyzed strains revealed 98% sequence identity. Analogous alignments of the intergenic regions upstream of *ureEF* showed 99% identity, while sequences upstream of *ureGD* exhibited 99% identity.

The sequence conservation of polypeptides comprising the urease system of both strains was examined further. The urease structural proteins UreA, UreB and UreC, as well as the accessory/regulatory proteins of the *Y. enterocolitica* strain Ye9N exhibit a high degree of amino acid sequence identity to those encoded by the corresponding open reading frames (ORFs) of *Y. enterocolitica* 8081 (98−100%) (data not shown).

Finally, using BLASTp searches to analyze the genomes of both *Y. enterocolitica* strains we identified a putative transcriptional regulator of the AraC family (289 aa) with homology to the regulator UreR identified in other ureolytic bacteria. Amino acid sequence alignment of this protein (strain 8081, GenBank: CAL12570) revealed 71% similarity (56% identity) to the urease operon transcriptional activator of *Serratia ficaria* (GenBank: SNW02772.1) and 43% similarity (22% identity) to UreR of *P. mirabilis* (GenBank: Z18752.1). Therefore, for consistency, this AraC-like protein of *Y. enterocolitica* will be termed UreR-like from here on. *In silico* analysis of this protein sequence using the MOTIF online tool (GenomeNet, Japan), identified helix-turn-helix motifs and an AraC-like binding domain.

The UreR-like protein of both strain 8081 (1B/O:8) (NCBI/GenBank: AM286415.1) and strain Ye9N (contig 5) is encoded by a gene located within a cluster of flagellar structural and assembly genes (downstream of the *fliT* gene and upstream of the gene encoding metal-dependent phosphohydrolase), and not in the vicinity of the *ure* cluster (Figure 3 and Supplementary Figure S1B). The nucleotide sequence identity of *ureR*-like genes of strains Ye9N and 8081 is 99% (and Supplementary Figure S1B), while that of the intergenic regions upstream of the *ureR*-like genes is 97%.

### OmpR Positively Affects the Expression of *ureABC*, *ureEF*, and *ureGD* in *Y. enterocolitica* Strain Ye9N

**FIGURE 4:**
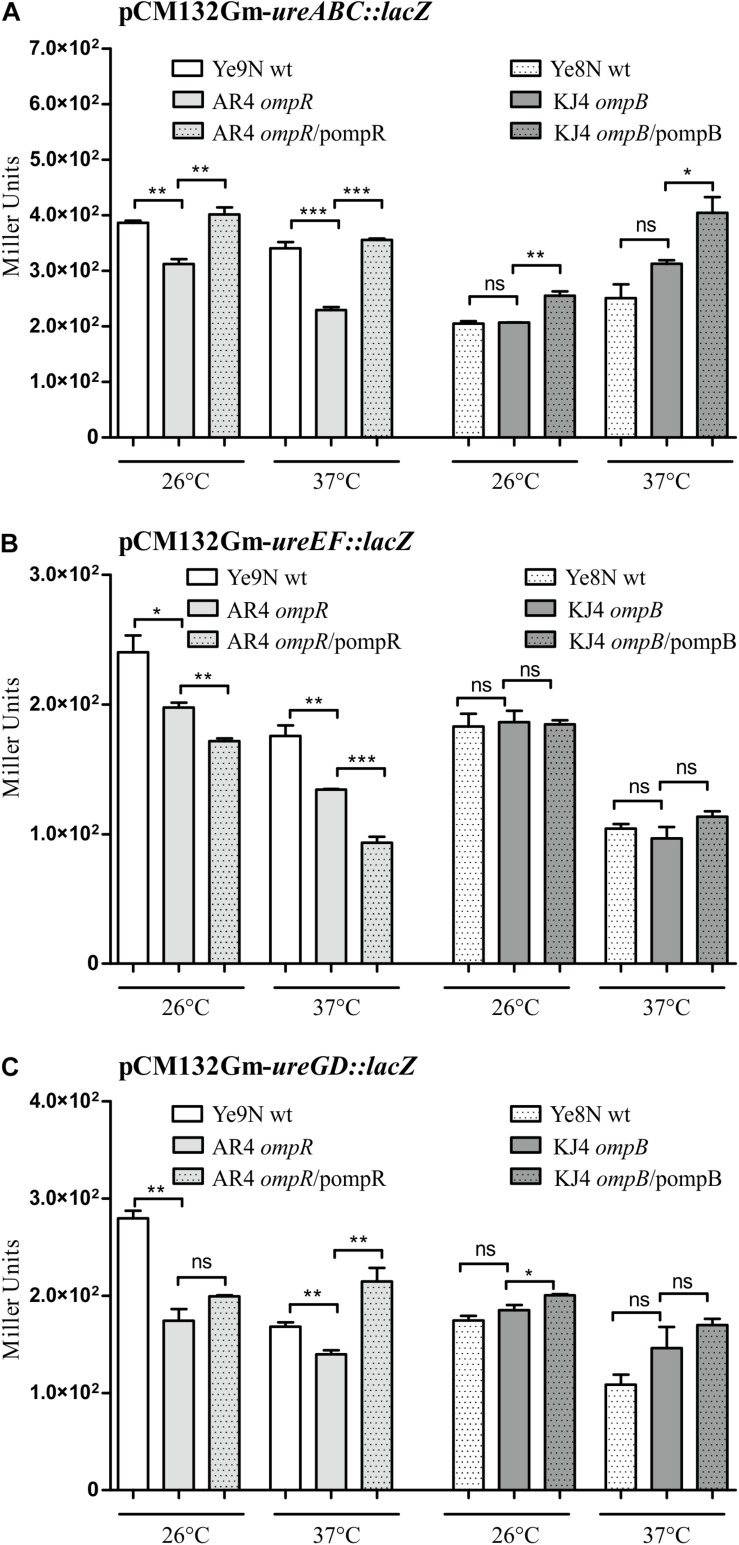
OmpR-dependent regulation of *ureABC*, *ureEF* and *ureGD* operon expression in *Y. enterocolitica*. β-galactosidase activity of strains carrying transcriptional fusions with *lacZ* expressed from plasmids pCM132Gm-*ureABC:lacZ* **(A)**, pCM132Gm-*ureEF:lacZ* **(B)** and pCM132Gm-*ureGD:lacZ* **(C)**, with or without OmpR, i.e., Ye9N (wt), AR4 (*ompR* mutant), AR4/pompR (complemented *ompR* mutant, *ompR*/pompR), Ye8N (wt), KJ4 (*ompB* mutant), KJ4/pompB (complemented *ompB* mutant, *ompB*/pompB). Strains were grown at 26°C or 37°C to stationary phase in LB medium. The data represent mean β-galactosidase activity values (Miller units) with the standard deviation from at least two independent experiments, each performed using at least triplicate cultures of each strain. Significance was calculated using Student’s unpaired *t*-test (***P ≤ 0.001; **P ≤ 0.01; and *P ≤ 0.05; ns, not significant, *P* > 0.05).

To examine the impact of regulator OmpR on the activity of urease operon promoters in different *Y. enterocolitica* strains, we constructed transcriptional fusions of the putative promoter sequences with the *lacZ* gene in plasmid pCM132Gm. The P_ureABC_, P_ureEF_, and P_ureGD_ promoters located upstream of the corresponding operons were identified using the bacterial promoter prediction program BPROM (Solovyev and Salamov, 2011). The three reporter gene constructs were introduced into the wild-type *Y. enterocolitica* strains Ye9N and Ye8N, and their respective OmpR-deficient derivatives AR4 (*ompR* mutant) and KJ4 (*ompB* mutant). The expression of the transcriptional fusions was quantified by measuring β-galactosidase activity in cells grown to stationary phase at 26°C or 37°C in LB medium (Figure 4). The activity of the *ureABC* promoter in the *ompR* mutant (AR4 strain) was lower than in the wild-type strain Ye9N at 26°C and 37°C (19 and 33% lower, respectively) (Figure 4A). To confirm positive OmpR-dependent regulation of this urease operon in strain Ye9N, a plasmid carrying a wild-type copy of the *ompR* gene (pompR in Figure 4) was used to complement the *ompR* mutation in strain AR4. This caused a statistically significant increase in the level of β-galactosidase activity from *ureABC:lacZ* at both tested temperatures, indicating that OmpR acts as a positive regulator of the operon encoding the three structural subunits of urease in this strain. The positive regulatory effect of OmpR was also observed for fusions carrying the promoter regions of the *ureEF* (Figure 4B) and *ureGD* (Figure 4C) operons. Reduced activity of *ureEF:lacZ* (18% and 24% decrease in activity) and *ureGD:lacZ* (38% and 17% decrease in activity) fusions was observed in the *ompR* mutant strain AR4 compared to the wild-type Ye9N at 26°C and 37°C, respectively. However, an effect of complementation with plasmid pompR was only detected for the reporter fusion *ureGD:lacZ* at 37°C. Interestingly, the OmpR-dependent regulation of urease operon transcription was not observed in *Y. enterocolitica* Ye8N, at either 26°C or 37°C. However, the β-galactosidase activity of *ureABC:lacZ* was increased in complemented strain KJ4/pompB at both temperatures. We concluded that OmpR positively and specifically influences *ure* expression in *Y. enterocolitica* strain Ye9N, but its role is less clear in strain Ye8N.

With regard to the influence of temperature on urease promoter activity, β-galactosidase production by each urease fusion was higher at 26°C compared to 37°C in *Y. enterocolitica* strain Ye9N: by 12% for *ureABC:lacZ*, 27% for *ureEF:lacZ* and 40% for *ureGD:lacZ*. In the case of strain Ye8N, temperature had no significant effect on expression of the *ureABC* reporter fusion. However, for *ureEF:lacZ* and *ureGD:lacZ*, lower β-galactosidase activity was observed at 37°C compared to 26°C. Furthermore, we found higher *ure* operon expression in strain Ye9N than in Ye8N at both tested temperatures. These data indicated OmpR- and temperature-dependent modulation of the *Y. enterocolitica* Ye9N *ure* operons that parallels urease activity (see Figure 1).

**FIGURE 5:**
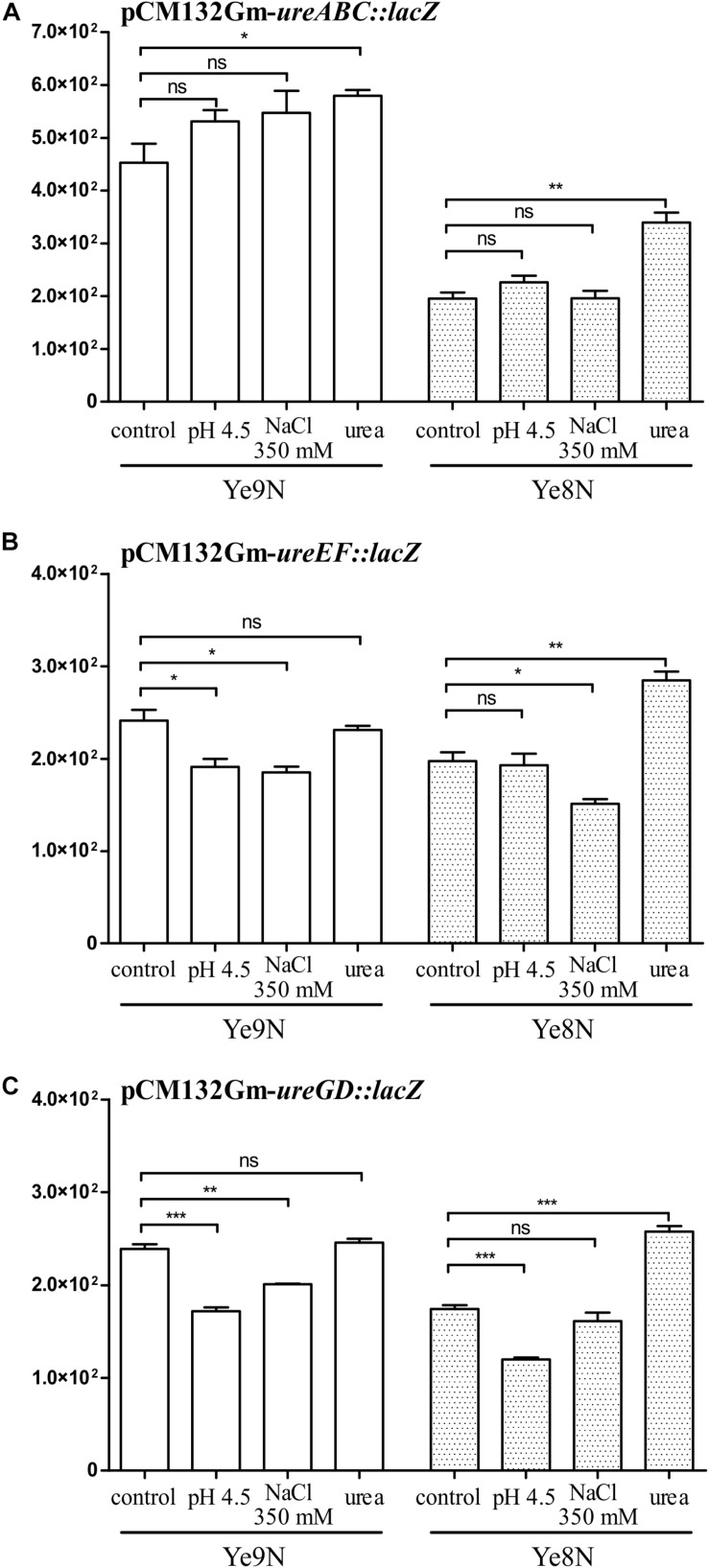
The effect of acid pH, high osmolarity and urea on the expression of the *ureABC*, *ureEF* and *ureGD* operons of *Y. enterocolitica*. β-galactosidase activity of strains Ye9N and Ye8N carrying transcriptional fusions with *lacZ* expressed from plasmids pCM132Gm-*ureABC:lacZ* **(A)**, pCM132Gm-*ureEF:lacZ* **(B)**, and pCM132Gm-*ureGD:lacZ* **(C)** was analyzed after incubating stationary phase cells at 26°C for 2 h in LB medium (86 mM NaCl, pH 7.0), LB adjusted to pH 4.5, LB supplemented with NaCl to a final concentration of 350 mM or LB containing 100 mM urea. The data represent mean β-galactosidase activity values (Miller units) with the standard deviation from at least two independent experiments, each performed using at least triplicate cultures of each strain. Significance was calculated using Student’s unpaired *t*-test (***P ≤ 0.001; **P ≤ 0.01; and *P ≤ 0.05; ns, not significant, *P* > 0.05).

The expression of many OmpR regulon genes in *E. coli* and *Salmonella* is osmoregulated and/or modulated in response to pH conditions (Chakraborty and Kenney, 2018). Next, we tested whether the expression of urease transcriptional units changes in response to high osmolarity and/or acidic conditions. Moreover, we were curious to see if urea could influence *ure* cluster gene expression. The effect of 350 mM NaCl, pH 4.5 and 100 mM urea on the β-galactosidase activity produced by *ure* operon:*lacZ* transcriptional fusions was tested in stationary phase cells of the wild-type *Y. enterocolitica* strains Ye9N and Ye8N grown at 26°C (Figure 5). No effect of high osmolarity (350 mM NaCl) or low pH (pH 4.5) was observed for the *ureABC:lacZ* fusion in strains Ye9N and Ye8N (Figure 5A). Only the addition of 100 mM urea caused a slight induction of *ureABC* expression in both tested strains. Measurements of β-galactosidase activity demonstrated that *ureEF:lacZ* expression was lower in strain Ye9N grown under acidic and high osmolarity conditions (Figure 5B). For strain Ye8N, a decrease in the expression of *ureEF:lacZ* was only observed in the presence of 350 mM NaCl. The addition of 100 mM urea significantly upregulated *ureEF:lacZ* expression only in strain Ye8N. The *ureEF* pattern of expression in response to high osmolarity, low pH and urea was also observed for the *ureGD:lacZ* fusion in strain Ye9N (Figure 5C). In strain Ye8N, significant changes in *ureGD:lacZ* expression were recorded in cells grown at pH 4.5 (downregulation) and in the presence of urea (upregulation).

### OmpR Regulates the Expression of a Gene Encoding a UreR-Like Putative Transcriptional Regulator

To examine whether *Y. enterocolitica ureR*-like expression responds to the presence of 100 mM urea, acidic conditions (pH 4.5) or high osmolarity (350 mM NaCl), and to test the influence of the regulator OmpR, we constructed a transcriptional fusion of the putative *ureR*-like promoter sequence identified by BPROM (Solovyev and Salamov, 2011) with the *lacZ* gene in vector pCM132Gm. This construct pCM132Gm- *ureR:lacZ* was introduced into the strains used to test the urease operon fusions, i.e., Ye9N and Ye8N and their OmpR-deficient derivatives, AR4 (*ompR* mutant) and KJ4 (*ompB* mutant).

**FIGURE 6:**
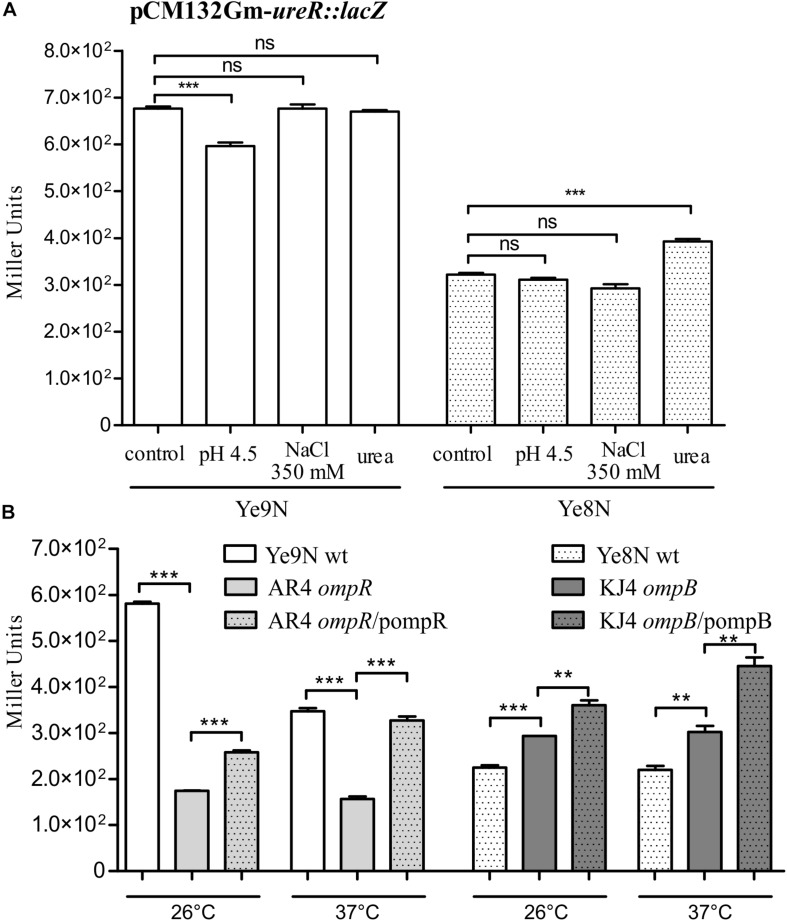
Analysis of *ureR*-like gene expression in *Y. enterocolitica* by measuring the β-galactosidase activity of strains carrying the plasmid pCM132Gm-*ureR:lacZ*. **(A)** The effect of acid pH, high osmolarity and urea on the *ureR*-like expression in *Y. enterocolitica* strains Ye9N and Ye8N was analyzed after incubating stationary phase cells at 26°C for 2 h in LB (86 mM NaCl, pH 7.0; control), LB adjusted to pH 4.5, LB supplemented with NaCl to a final concentration of 350 mM or LB containing 100 mM urea. **(B)** OmpR-dependent regulation of *ureR-*like gene expression in Ye9N (wt), AR4 (*ompR* mutant), AR4/pompR (complemented *ompR* mutant, *ompR*/pompR), Ye8N (wt), KJ4 (*ompB* mutant) and KJ4/pompB (complemented *ompB* mutant, *ompB*/pompB). Strains were grown at 26°C or 37°C to stationary phase in LB medium. The data represent mean β-galactosidase activity values (Miller units) with the standard deviation from at least two independent experiments, each performed using at least triplicate cultures of each strain. Significance was calculated using Student’s unpaired *t*-test (***P ≤ 0.001 and **P ≤ 0.01; ns, not significant, *P* > 0.05).

As shown in Figure 6A, high osmolarity had no effect on *ureR:lacZ* expression in either of the strains Ye9N or Ye8N. A 2-h exposure to an acidic environment resulted in decreased *ureR-like* expression in the strain Ye9N. A slight stimulatory effect of urea (1.2-fold) was observed, but only in the strain Ye8N, which parallels the induction of *ure* gene expression by urea seen in this strain.

The *ureR:lacZ*β-galactosidase activity detected in the *ompR* mutant (strain AR4) was lower than that measured in the wild-type strain Ye9N at 26°C or 37°C (3.3- and 2.2-fold lower, respectively), and this effect was complemented by the wild-type *ompR* allele provided *in trans* (Figure 6B). These data suggested that OmpR is a positive regulator of the *ureR*-like gene in strain Ye9N. Interestingly, we found that the absence of the *ompB* in strain KJ4 resulted in the upregulation of *ureR-*like gene expression at both tested temperatures (by 1.3-fold at 26°C and 1.4-fold at 37°C). However, the addition of plasmid-encoded *ompB* to strain KJ4 did not complement the *ompB* deletion and even upregulated the *ureR*-like gene expression. These findings indicated that OmpR has the opposite regulatory effect on *ureR-*like gene expression in the two tested *Y. enterocolitica* strains, i.e., positive in Ye9N and negative in Ye8N.

### OmpR Binds Directly to the *ureABC*, *ureEF* and *ureR*-Like Regulatory Regions

**FIGURE 7:**
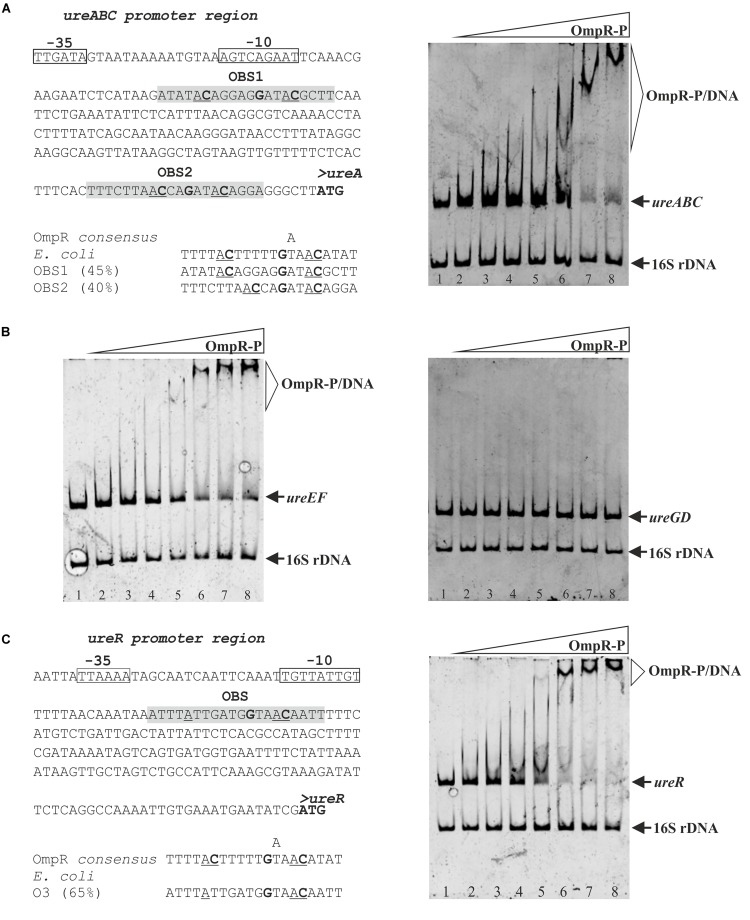
Interaction of OmpR with the *ureABC*, *ureEF*, *ureGD* and *ureR*-like regulatory regions of *Y. enterocolitica*. (**A**, left panel) Putative OmpR-binding sites (OBS) in the promoter region of the *ureABC* operon of *Y. enterocolitica* identified based on similarity to the *E. coli* consensus sequence (% identity values are shown). (**A**, right panel) Binding of phosphorylated OmpR to the *ureABC* regulatory region examined using an EMSA. **(B)** Binding of phosphorylated OmpR to the *ureEF* and *ureGD* regulatory regions examined using EMSAs. (**C**, left panel) Putative OmpR-binding site in the promoter of the *ureR*-like gene of *Y. enterocolitica* (% identity to the *E. coli* consensus sequence is shown) (**C**, right panel) interaction of OmpR with the *ureR*-like regulatory region examined using an EMSA. In the *in silico* analysis (**A,C**, left panels) the start codons are shown in bold. The likely –10 and –35 promoter elements are marked. The potential OBS in the promoter regions of *ureABC* and the *ureR-*like gene are shaded gray with the central GXXAC motifs marked. In the EMSAs, different concentrations of OmpR-P were incubated with DNA fragments representing the *ureABC* (312 bp), *ureEF* (385 bp), *ureGD* (375 bp), and *ureR-*like (363 bp) regulatory regions. A 16S rDNA fragment (211 bp) was included in each reaction as a non-specific binding control. The reaction mixtures contained 0.05 pmol of each fragment and increasing amounts of OmpR-P: for the *ure* operon fragments **(A,B)**, lanes 1 – no protein, lanes 2 – 0.93 μM, lanes 3 – 1.24 μM, lanes 4 – 1.85 μM, lanes 5 – 2.47 μM, lanes 6 – 3.71 μM, lanes 7 – 4.33 μM, and lanes 8 – 4.95 μM; for the *ureR*-like fragment **(C)**, lane 1 – no protein, lane 2 – 0.31 μM, lane 3 – 0.46 μM, lane 4 – 0.62 μM, lane 5 – 0.77 μM, lane 6 – 0.93 μM, lane 7 – 1.85 μM, and lane 8 – 2.78 μM. The identities of the bands resolved by electrophoresis on native 4.2% polyacrylamide gels are indicated.

We next tested whether OmpR may influence *ure* operon expression directly. *In silico* analysis of the regulatory sequence of *ureABC* (Figure 7A left panel) identified two 20-bp putative OmpR-binding sites (OBS), with 45% and 40% homology to the *E. coli* OmpR consensus sequence [TTTTACTTTTTG(A/T)AACATAT] (Maeda et al., 1991; Harlocker et al., 1995). No potential OBS were recognized in either the *ureEF* or the *ureGD* regulatory regions.

To determine whether OmpR could modulate *ureABC*, *ureEF* and *ureGD* expression by directly binding to the regulatory regions of these operons, we employed electrophoretic mobility shift assays (EMSAs) (Figure 7A right panel and 7B). DNA fragments containing the regulatory regions of the three urease operons were amplified by PCR and used in EMSAs with increasing concentrations of phosphorylated OmpR-His_6_ (OmpR-P). A specific shifted DNA-protein complex was formed by the interaction between the *ureABC* regulatory region fragment and phosphorylated OmpR (Figure 7A right panel). We observed a stepwise increase in shifted bands of decreasing mobility as the OmpR concentration was increased from 2.47 μM up to 4.95 μM, suggesting that more than one binding site is present. Although no potential OBS were identified in the *ureEF* promoter sequence, phosphorylated OmpR also interacted with this regulatory region and a slower migrating DNA-protein band appeared at an OmpR-P concentration of 2.47 μM (Figure 7B left panel). As shown in Figure 7B right panel, OmpR-P did not interact with the regulatory region of *ureGD*, which is consistent with the apparent absence of OBS. OmpR was unable to bind the negative control 16S rDNA fragment included in each binding reaction.

Following the demonstration that OmpR regulates *ureR*-like gene expression in *Y. enterocolitica*, we hypothesized that this regulator may do so directly. One potential OBS was identified in the *ureR*-like gene regulatory region by *in silico* analysis (Figure 7C left panel). The predicted OBS exhibits high sequence identity (65%) to the consensus OmpR-binding sequence of *E. coli*. An EMSA was then used to examine the ability of the phosphorylated OmpR regulator to bind to the *ureR*-like regulatory region *in vitro* (Figure 7C right panel). This analysis revealed the appearance of specific slower migrating OmpR*-*DNA complexes concomitantly with the disappearance of the *ureR*-like regulatory region fragment band. In comparison with the EMSAs performed using the *ureABC* and *ureEF* regulatory region fragments (Figures 7A,B), the OmpR-P/*ureR*-like complexes were formed at a lower OmpR concentration (i.e., at 0.77 μM rather than 2.47 μM) suggesting higher binding affinity of OmpR for the *ureR*-like target.

## Discussion

This study started by examining the impact of OmpR, the response regulator of the two-component signal transduction system EnvZ/OmpR, on the cytoplasmic proteome of *Y. enterocolitica* Ye9N (bio-serotype 2/O:9) at 26°C (optimum temperature for growth) and 37°C (the body temperature of mammalian hosts). The results of the proteomic analysis indicated that temperature and OmpR affect (both positively and negatively) the production of a number of proteins that serve a variety of functions. OmpR was found to be involved in the expression of some regulatory proteins, including HU-alpha, sigma 54 and TF (Dillon and Dorman, 2010; Hoffmann et al., 2010; Danson et al., 2019), as well as effector proteins of the Type III Secretion System, the major virulence factors of pathogenic *Y. enterocolitica* (Cornelis, 2002). Thus, OmpR appears to influence the adaptive and virulence properties of *Y. enterocolitica*.

Interestingly, differential analysis of the cytoplasmic proteome at 26°C and 37°C identified components of the urease system among the set of temperature-regulated proteins. The levels of UreC, UreE and UreG were reduced at 37°C compared to 26°C, which is in line with the findings of a study carried out on the low pathogenic *Y. enterocolitica* strain W227 serotype O:9 (De Koning-Ward and Robins-Browne, 1997). It was suggested previously that higher expression of urease at moderate temperature, might be important for the adaptive ability of *Y. enterocolitica* during the earliest stage of pathogenesis and assists passage through the acidic environment of the stomach (Young et al., 1996; De Koning-Ward and Robins-Browne, 1997). It has also been proposed that low-temperature-induced expression of the *ure* genes observed in strain W22703 (bio-serotype 2/O:9) may play a role in the adaptive abilities of low-pathogenic *Y. enterocolitica* strains during bacteria-insect interactions (Heermann and Fuchs, 2008; Fuchs et al., 2011).

A significant finding of our quantitative proteomic analysis is that the regulator OmpR positively affects the abundance of components of the *Y. enterocolitica* urease system at both tested temperatures (26°C and 37°C). The impact of the OmpR regulator on urease expression was examined in more detail in *Y. enterocolitica* strain Ye9N of bio-serotype 2/O:9 and strain Ye8N of bio-serotype 1B/O:8 (derivative of clinical isolate 8081). The high-level pathogenic strain 8081, isolated in the United States, is the most studied *Y. enterocolitica* strain with respect to its virulence factors and their contribution to pathogenesis in mammals (Portnoy et al., 1981). The low-level pathogenic strain Ye9N (bio-serotype 2/O:9), isolated in Poland, has been the research object of many studies, especially concerning the role of OmpR in regulating the virulence properties and adaptive abilities of *Y. enterocolitica* (Brzostek et al., 2003, 2007; Raczkowska et al., 2011, 2015; Skorek et al., 2013; Nieckarz et al., 2016, 2017).

Upon analyzing these two strains in parallel, higher levels of urease activity were observed in Ye9N than in Ye8N, and in cells grown at 26°C compared to 37°C. More interestingly, OmpR exerted a positive impact on urease activity in the strain Ye9N grown at 26°C. At 37°C this effect was less obvious since a wild-type copy of *ompR* expressed from a plasmid did not complement the decrease in urease activity caused by the *ompR* mutation. This suggests that a more complex regulatory mechanism influences the production/activity of urease at 37°C in the presence of OmpR supplied *in trans*. No positive impact of OmpR on urease activity was observed in strain Ye8N, irrespective of the growth temperature. Thus, OmpR-dependent regulation of urease activity seems to be characteristic of particular *Y. enterocolitica* strains. Phenotypic variations among closely related strains of *Y. enterocolitica*, even those belonging to the same bio-serotype, have been highlighted previously (Garzetti et al., 2012).

Other phenotypic studies of *Y. enterocolitica* strains Ye9N and Ye8N have demonstrated that OmpR increases, to a varying degree, the survival of both strains in acidic conditions. Since these acid survival tests were not performed in the presence of urea, the observed differences were not related to urease activity and this suggested distinct roles for OmpR in other acid-tolerance mechanisms operating in these bacteria at 26°C and 37°C. Intra-species differences in acid survival are not specific to *Y. enterocolitica* strains, since variability in acid resistance has also been reported among isolates of *Salmonella* Typhimurium (Berk et al., 2005). It may be concluded that the role of OmpR in acid survival of *Y. enterocolitica* strains is complex and involves urease-dependent and -independent mechanisms.

In the course of this study we verified the hypothesis that OmpR directly regulates expression of the *ure* operons in *Y. enterocolitica*. Firstly, promoter-*lacZ* reporter gene fusion assays revealed that *ure* operon expression is higher in strain Ye9N than in Ye8N, and higher at 26°C than at 37°C, which is consistent with the proteomic data and urease activity tests. Interestingly, in the case of strain Ye8N, temperature significantly affected expression of *ureEF:lacZ* and *ureGD:lacZ* reporter fusions but not *ureABC:lacZ*. Thus, the overall thermoregulation of urease activity depends on the expression of the structural genes. Secondly, we demonstrated that OmpR acts as a positive regulator of the *ureABC*, *ureEF* and *ureGD* operons in strain Ye9N, both at 26°C and 37°C. The OmpR-dependent regulation of urease cluster transcription was not observed in strain Ye8N, even though the *ure* loci of both strains exhibit high nucleotide sequence identity (98%). It has been relatively well established that although *Y. enterocolitica* strains possess the same virulence factors, their expression profiles can differ significantly (Schmühl et al., 2019). This may be the consequence of the finding that even small alterations in the content and organization of the genetic information may alter the expression pattern of virulence genes and their regulators in response to environmental factors (Uliczka et al., 2011; Erhardt and Dersch, 2015).

The generally accepted mechanism of OmpR activation in bacteria is phosphorylation of this regulator by its partner kinase EnvZ in response to high osmolarity and low pH (Russo and Silhavy, 1990; Hoch and Silhavy, 1995; Schwan et al., 2002; Rentschler et al., 2013). This study has revealed that OmpR positively regulates expression of the *ureABC*, *ureEF* and *ureGD* operons in *Y. enterocolitica* strain Ye9N. However, no effect of high osmolarity (350 mM NaCl) or low pH (pH 4.5) was observed in the case of the *ureABC* operon encoding the urease subunits. We presume that a combination of environmental signals or some other stimuli activate OmpR to regulate *ureABC* expression. It was previously suggested that the activation of *Y. enterocolitica* urease by low pH is not due to increased *ure* gene expression (Young et al., 1996). Interestingly, the acid-induction of urease cluster transcriptional units has been demonstrated in *Y. pseudotuberculosis* strain YPIII, and OmpR was identified as playing a key role in this regulation (Hu et al., 2009). However, *Y. pseudotuberculosis* differs considerably from *Y. enterocolitica* in genomic content and the degree of pathogenicity (Galindo et al., 2011) and it is distantly related from an evolutionary point of view (McNally et al., 2016; Tan et al., 2016). Moreover, in contrast to *Y. enterocolitica* O:9, urease does not seem to be involved in the virulence of *Y. pseudotuberculosis* (Riot et al., 1997).

Despite the failure to detect induction by acidic conditions, slightly higher expression of *ureABC* in strain Ye9N and of all *ure* operons in strain Ye8N were observed in the presence of urea. The induction of urease structural genes by urea has been reported in *Proteus* and *Providentia* species, with levels 5- to 25-fold greater than those in uninduced cultures (Mobley et al., 1995). In these species, the urease induction is mediated by the transcriptional regulator UreR, which is encoded by a gene located upstream of the *ureDABCEFG* cluster (Mobley et al., 1995). No such *ureR*-like gene is present in the vicinity of the *Y. enterocolitica ure* cluster. Through comparative bioinformatic analysis we identified an AraC-like protein with 71% similarity (56% identity) to the urease operon transcriptional activator of *Serratia ficaria.* However, amino acid sequence alignment with UreR of *P. mirabilis* revealed only 43% similarity (22% identity). In this study, we employed transcriptional fusion analysis and an EMSA to show that OmpR directly regulates the expression of this *ureR*-like gene in *Y. enterocolitica* strains Ye9N and Ye8N. Interestingly this gene is regulated in the opposite manner in these strains (positively in Ye9N and negatively in Ye8N), but the nature of this reciprocal regulation is unknown. Possibly, the strain-specific differences in OmpR-dependent regulation of the urease cluster that we observed are due to its opposite effect on the expression of the UreR-like regulator. The function of this putative *Y. enterocolitica* UreR regulator requires further investigation.

Finally, in the course of this study we verified the hypothesis that OmpR directly regulates the expression of the *ure* operons in *Y. enterocolitica* strain Ye9N. Putative OBS were identified in the regulatory regions of the studied transcriptional units based on similarity to the consensus sequence described for OmpR of *E. coli* (Maeda et al., 1991; Harlocker et al., 1995). The results of EMSAs demonstrated that phosphorylated OmpR can specifically bind to the *ureABC* and *ureEF* promoter regions, which suggests direct regulation. In contrast, OmpR-dependent regulation of *ureGD* expression seems to be indirect.

## Conclusion

In summary, this study has revealed different roles for OmpR in the regulation of urease expression in two *Y. enterocolitica* strains representing different bio-serotypes with distinct pathogenic abilities. Thus, it has increased our understanding of the function of OmpR in the survival of *Y. enterocolitica* under conditions where the activity of urease is crucial.

At this time it cannot be concluded that these differences in the role of OmpR are a common characteristic of other *Y. enterocolitica* strains of these bio-serotypes. Further comparative analysis of multiple representatives of particular bio-serotypes is required to determine whether OmpR-dependent urease expression is a strain- or bio-serotype-specific adaptation to increase survival in the natural environment, or within certain hosts or particular host niches.

## Data Availability Statement

All datasets generated for this study are included in the article/Supplementary Material.

## Author Contributions

MN designed the study and prepared the figures. MN, PK, and KJ performed the experiments. MN, AR, and KB analyzed the data. MN and KB wrote the manuscript and provided the financial support.

## Conflict of Interest

The authors declare that the research was conducted in the absence of any commercial or financial relationships that could be construed as a potential conflict of interest.

## Footnotes

1 http://www.ncbi.nlm.nih.gov/BLAST/

2 https://www.genome.jp/tools/motif/

## Supplementary Material

FIGURE S1 Characterization of the *ure* gene cluster and *ureR*-like gene in *Y. enterocolitica* strains: Ye9N (2/O:9), (contig 39 – NCBI/GenBank: JAALCX010000039 and contig 5 – NCBI/GenBank: JAALCX010000005) and 8081 (1B/O:8), (NCBI/GenBank: AM286415). Nucleotide sequence alignments of the *ure* gene cluster composed of the seven ORFs encoding the structural (UreABC) and accessory (UreEFGD) proteins of the urease system, and the intergenic regions **(A)** and the *ureR*-like gene encoding the putative UreR-like transcriptional regulator **(B)**. The Needleman-Wunsch algorithm was used to draw the alignment. Percentage identity of the DNA sequences in the alignments is shown. The likely −10 and −35 promoter elements, indicated by BPROM software, are underlined. The start codons are marked in green and the stop codons are marked in gray. The *ure* gene cluster is flanked upstream by the gene encoding a hypothetical protein and downstream by the *yut* gene. The *ureR*- like gene is localized between the *fliT* gene and the gene encoding a metal-dependent phosphohydrolase. The locus tags in the Ye9N strain are indicated.

TABLE S1 Strains and plasmids used in this study.

TABLE S2 Oligonucleotide primers used in this study. The table shows the sequences of primers used for cloning, the construction of transcriptional fusions, and to generate regulatory region fragments for EMSAs.
