# Supplementary material for: Urease Expression in Pathogenic Yersinia enterocolitica Strains of Bio-Serotypes 2/O:9 and 1B/O:8 Is Differentially Regulated by the OmpR Regulator
Source: Front Microbiol. 2020 Apr 8;11:607. doi: 10.3389/fmicb.2020.00607 (PMC7156557; doi:10.3389/fmicb.2020.00607)
Supplement: FIGURE S1 — Characterization of the ure gene cluster and ureR-like gene in Y. enterocolitica strains: Ye9N (2/O:9), (contig 39 – NCBI/GenBank: JAALCX010000039 and contig 5 – NCBI/GenBank: JAALCX010000005) and 8081 (1B/O:8), (NCBI/GenBank: AM286415). Nucleotide sequence alignments of the ure gene cluster composed of the seven ORFs encoding the structural (UreABC) and accessory (UreEFGD) proteins of the urease system, and the intergenic regions (A) and the ureR-like gene encoding the putative UreR-like transcriptional regulator (B). The Needleman-Wunsch algorithm was used to draw the alignment. Percentage identity of the DNA sequences in the alignments is shown. The likely −10 and −35 promoter elements, indicated by BPROM software, are underlined. The start codons are marked in green and the stop codons are marked in gray. The ure gene cluster is flanked upstream by the gene encoding a hypothetical protein and downstream by the yut gene. The ureR- like gene is localized between the fliT gene and the gene encoding a metal-dependent phosphohydrolase. The locus tags in the Ye9N strain are indicated. [file Image_1.pdf]

(A)

The urease locus
identities 6385/6515(98%)

```
              gene encoding hypothetical protein
              (locus_tag=G5S39_RS14435)
              codon stop
Ye9N   1      TAATGCCCTATAGCCTGTCGCAGTTATTTGTGATGGGAAGGGACAATGGTCGTCGTTTTT  60
              |||||||||||||||||||||||||||||| |||||||||||||||| ||| ||||||||
8081   1      TAATGCCCTATAGCCTGTCGCAGTTATTTGCGATGGGAAGGGACAATAGTCATCGTTTTT  60
                                                                -35
Ye9N   61     AGTCTGTTTCGCGATATTAAAGGTTACAGAATAAATATCTGGCAGCAGATTGATAGTAAT  120
              ||||||||||||||||||||||||||||||||||||||||||||||||||||||||||||
8081   61     AGTCTGTTTCGCGATATTAAAGGTTACAGAATAAATATCTGGCAGCAGATTGATAGTAAT  120
                           -10
Ye9N   121    AAAAATGTAAAGTCAGAATTCAAACGAAGAATCTCATAAGATATACAGGAGGATACGCTT  180
              |||||||||||||||||||||||||||||||||||| ||| |||||||||||||||||||
8081   121    AAAAATGTAAAGTCAGAATTCAAACGAAGAATCTCACAAGCTATACAGGAGGATACGCTT  180

Ye9N   181    CAATTCTGAAATATTCTCATTTAACAGGCGTCAAAACCTACTTTTATCAGCAATAACAAG  240
              |||||||||||||||||||||||||||||||||||| |||||||||||||||||||||||
8081   181    CAATTCTGAAATATTCTCATTTAACAGGCGTCAAAATCTACTTTTATCAGCAATAACAAG  240

Ye9N   241    GGATAACCTTTATAGGCAAGGCAAGTTATAAGGCTAGTAAGTTGTTTTTCTCACTTTCAC  300
              ||||||||||||||||||||||||||||||||||||||| ||||||||||||||||||||
8081   241    GGATAACCTTTATAGGCAAGGCAAGTTATAAGGCTAGTAGGTTGTTTTTCTCACTTTCAC  300
                                         >ureA (locus_tag=G5S39_RS14440)
Ye9N   301    TTTCTTAACCAGATACAGGAGGGCTTATGCAGCTCACCCCAAGAGAAGTTGAAAAGCTCA  360
              ||||||||||||||||||||||||||||||||||||||||||||||||||||||||||||
8081   301    TTTCTTAACCAGATACAGGAGGGCTTATGCAGCTCACCCCAAGAGAAGTTGAAAAGCTCA  360

Ye9N   361    TGATCTACACGCTGTCTGATGTGGCGTTCAAACGCAAAGCGCGTGGCTTGAAACTCAATT  420
              ||||||||||||||||||||||||||||||||||||||||||||||||||||||| ||||
8081   361    TGATCTACACGCTGTCTGATGTGGCGTTCAAACGCAAAGCGCGTGGCTTGAAACTTAATT  420

Ye9N   421    ATCCGGAAGCCGTTTCTATTATCACAGTGACTGCAATGGAAGGGGCCAGAGATGGCAAAT  480
              ||||||||||||||||||||||||||||||||||||||||||||||||||||||||||||
8081   421    ATCCGGAAGCCGTTTCTATTATCACAGTGACTGCAATGGAAGGGGCCAGAGATGGCAAAT  480

Ye9N   481    CCGTAGAGGATGTGATGAAAGAAGCCAGTAAAGTTCTCACAAAAGATGATGTGATGGACG  540
              ||||||||||||||||||||||||||||||||||||||||||||||||||||||||||||
8081   481    CCGTAGAGGATGTGATGAAAGAAGCCAGTAAAGTTCTCACAAAAGATGATGTGATGGACG  540

Ye9N   541    GGGTGGCTGATCTGATTCCGAATGTTCAGGTTGAAGCCATTTTTACCGACGGCAGTCGTT  600
              ||||||||||||||||||||||||||||||||||||||||||||||||||||||| ||||
8081   541    GGGTGGCTGATCTGATTCCGAATGTTCAGGTTGAAGCCATTTTTACCGACGGCAGCCGTT  600
                                         codon stop
Ye9N   601    TGGTCACGGTGCACGACCCTATCAAATGAGTGACGGCAGCATGCGAGTAAAACCTGATCT  660
              ||||||||||||||||||||||||||||||||||||||||||||||||||||||||||||
8081   601    TGGTCACGGTGCACGACCCTATCAAATGAGTGACGGCAGCATGCGAGTAAAACCTGATCT  660
                                   >ureB (locus_tag=G5S39_RS14445)
Ye9N   661    GAACATTGCAGAGGATTAAAGCATGAGCACAAAGACAAATAGCACCAAAGCAACGAGTGA  720
              ||||||||||||||||||||||||||||||||||||||||||||||||||||||||||||
8081   661    GAACATTGCAGAGGATTAAAGCATGAGCACAAAGACAAATAGCACCAAAGCAACGAGTGA  720

Ye9N   721    AAAGACAGATAGCTTAAAGACAAATAGTGGCACTAAATCAAGTGCAGGTTATTCTGAACA  780
              ||||||||||||||||||||||||||| ||||||||||||||||||||||||||||||||
8081   721    AAAGACAGATAGCTTAAAGACAAATAGAGGCACTAAATCAAGTGCAGGTTATTCTGAACA  780

Ye9N   781    AAATACTCCACTCGGTGGCTGCATTTTAGCCGATACTCCGATCACCTTTAATGAAAATAA  840
              |||||| ||||||||||| |||||||||||||||||| ||||||||||||||||||||||
8081   781    AAATACCCCACTCGGTGGTTGCATTTTAGCCGATACACCGATCACCTTTAATGAAAATAA  840

Ye9N   841    GCCTGTTACCAAAGTGAAAGTTCGCAACACAGGTGACCGGCCTATTCAGGTGGGGTCACA  900
              |||||||||||||||||||||||||||||| ||||||||||| ||||||||||| |||||
8081   841    GCCTGTTACCAAAGTGAAAGTTCGCAACACCGGTGACCGGCCGATTCAGGTGGGTTCACA  900

Ye9N   901    TTTCCATTTCTTTGAAGTTAATCGCGCACTGGAATTTGACCGCGCGGCGGCTTACGGAAA  960
              |||||| |||||||||| |||||| |||||||| |||||||| || ||||||||||||||
8081   901    TTTCCACTTCTTTGAAGCTAATCGGGCACTGGAGTTTGACCGTGCTGCGGCTTACGGAAA  960

Ye9N   961    AAGACTGAATATCTCTTCAACCACCGCAATCCGTTTTGAACCCGGTGATGAAACCGAAGT  1020
              ||||||||||||||||||||||||||| ||||||||||||||||||||||||||||||||
8081   961    AAGACTGAATATCTCTTCAACCACCGCCATCCGTTTTGAACCCGGTGATGAAACCGAAGT  1020
```

```
Ye9N   1021  TCCGCTGATTCCTTTTGGTGGTAAGCAAACACTGTATGGCTTTAACAACCTGGTGGATGG  1080
             |||||||||||||||||||||||||||||| |||||||| ||||| ||||||||||||||
8081   1021  TCCGCTGATTCCTTTTGGTGGTAAGCAAACCCTGTATGGTTTTAATAACCTGGTGGATGG  1080

Ye9N   1081  TTGGACTGGTGAAGGCGTCGTTCCCAATAGCGAACGTCCGGATAAGCTAGAGGCTATTCG  1140
             ||||||||||||||||||||||||||||||||||||||||||||||||||||||||||||
8081   1081  TTGGACTGGTGAAGGCGTCGTTCCCAATAGCGAACGTCCGGATAAGCTAGAGGCTATTCG  1140
                                                codon stop
Ye9N   1141  TCGTGCGGCTGAGCGTGGCTTCAAATCGTCTAAATGACACCTTATCTCACTAAAAATTAT  1200
             |||||| |||||||||||||||||||||||||||||||||||||||||||||||||||||
8081   1141  TCGTGCAGCTGAGCGTGGCTTCAAATCGTCTAAATGACACCTTATCTCACTAAAAATTAT  1200

Ye9N   1201  CGAAATTCGCATACGTGTAATTAGCTATAACCAATAGATTTCAAGTTTCAGGAAGGCGGC  1260
             ||||||||||||||||||||||||||||||||||||||||||||||||||||||||||||
8081   1201  CGAAATTCGCATACGTGTAATTAGCTATAACCAATAGATTTCAAGTTTCAGGAAGGCGGC  1260

Ye9N   1261  AAACGAGAGTCCCGATGAGCTAACACACCTGCAACTTGAAAGATGAAGGTTACGCGTACC  1320
             ||||||||||||||||||||||||||||||||||||||||||||||||||||||||||||
8081   1261  AAACGAGAGTCCCGATGAGCTAACACACCTGCAACTTGAAAGATGAAGGTTACGCGTACC  1320
                                &gt;ureC (locus_tag=G5S39_RS14450)
Ye9N   1321  GGAAAAAGAAAGGAGCGACAGATGCCTCAAATTTCTCGGCAAGAATACGCGGGTCTATTT  1380
             ||||||||||||||||||||||||||||||||||||||||||||||||||||||||||||
8081   1321  GGAAAAAGAAAGGAGCGACAGATGCCTCAAATTTCTCGGCAAGAATACGCGGGTCTATTT  1380

Ye9N   1381  GGCCCAACGACTGGCGATAAAATCCGTTTGGGTGACACCAATCTATTTATCGAAATCGAA  1440
             ||||||||||||||||||||||||||||||||||||||||||||||||||||||||||||
8081   1381  GGCCCAACGACTGGCGATAAAATCCGTTTGGGTGACACCAATCTATTTATCGAAATCGAA  1440

Ye9N   1441  AAAGACCTGCGTGGATATGGTGAAGAGTCGGTTTACGGTGGGGGTAAGTCATTGCGTGAC  1500
             |||||||||||||||||||||||||||||||||||||||||||| |||||||||||||||
8081   1441  AAAGACCTGCGTGGATATGGTGAAGAGTCGGTTTACGGTGGGGGCAAGTCATTGCGTGAC  1500

Ye9N   1501  GGGATGGGCGCGAATAACCATCTGACCCGCGATAACGGTGTACTGGATTTAGTCATAACC  1560
             ||||||||||||||||||||||||||||||||||||||||||||||||||||||||||||
8081   1501  GGGATGGGCGCGAATAACCATCTGACCCGCGATAACGGTGTACTGGATTTAGTCATAACC  1560

Ye9N   1561  AACGTCACTATTGTTGATGCTCGTTTAGGGGTTATCAAAGCCGACGTCGGTATCCGTGAT  1620
             ||||||||||||||||||||||||||||||||||||||||||||||||||||||||||||
8081   1561  AACGTCACTATTGTTGATGCTCGTTTAGGGGTTATCAAAGCCGACGTCGGTATCCGTGAT  1620

Ye9N   1621  GGTAAAATTGCTGGTATTGGTAAAAGTGGCAACCCAGGCGTGATGGACGGAGTTACACCT  1680
             ||||||||||| ||||| ||||||||||| ||||| || |||||||| || || || || 
8081   1621  GGTAAAATTGCCGGTATCGGTAAAAGTGGTAACCCTGGGGTGATGGATGGTGTCACTCCC  1680

Ye9N   1681  GGACTGGTGGTGGGGGTCAGTACCGATGCCATTTCCGGTGAACATTTGATTCTCACTGCC  1740
             ||  ||||||| || || || |||||||| ||||||||||||||||||||||||||||||
8081   1681  GGCATGGTGGTAGGCGTTAGCACCGATGCTATTTCCGGTGAACATTTGATTCTCACTGCC  1740

Ye9N   1741  GCCGGTATTGATACTCATATTCACTTAATTTCTCCACAACAGGCTTACCATGCCTTATCC  1800
             |||||||||||||  || ||||||||||| || ||||||||||||||||||||  | || 
8081   1741  GCCGGTATTGATAGCCACATTCACTTAATCTCCCCACAACAGGCTTACCATGCGCTCTCT  1800

Ye9N   1801  AATGGCGTGGCGACTTTCTTTGGCGGTGGGATTGGCCCAACTGATGGCACCAACGGGACG  1860
             ||||||||||| || ||||| || || |||||||||||||| |||||||| ||||| || 
8081   1801  AATGGCGTGGCAACCTTCTTCGGTGGCGGGATTGGCCCAACCGATGGCACTAACGGAACC  1860

Ye9N   1861  ACAGTCACTCCCGGCCCGTGGAACATTCGCCAGATGCTGCGCTCAGTTGAAGGGCTGCCG  1920
             || || || || ||||| ||||| ||||||||||||||||||||||||||||||||||||
8081   1861  ACCGTTACCCCTGGCCCTTGGAATATTCGCCAGATGCTGCGCTCAGTTGAAGGGCTGCCG  1920

Ye9N   1921  GTCAACGTGGGTATTCTGGGTAAAGGTAACTCTTACGGCCGTGGCCCGTTGTTAGAACAG  1980
             |||||||||||||||||||||||||||||||||||||||||||||||| |||| ||||||
8081   1921  GTCAACGTGGGTATTCTGGGTAAAGGTAACTCTTACGGCCGTGGCCCGCTGTTGGAACAG  1980

Ye9N   1981  GCGATTGCCGGTGTTGTCGGCTATAAAGTCCACGAAGACTGGGGCGCGACAGCCAATGCC  2040
             ||||||||||||||||||||||||||||||||||||||||||||||||||||||||||||
8081   1981  GCGATTGCCGGTGTTGTCGGCTATAAAGTCCACGAAGACTGGGGCGCGACAGCCAATGCC  2040

Ye9N   2041  CTGCGCCATTCATTACGGATGGCGGATGAAATGGATATTCAGGTTTCCGTACATACCGAC  2100
             ||||||||||||||||||||||||||||||||||||||||||||||||||||||||||||
8081   2041  CTGCGCCATTCATTACGGATGGCGGATGAAATGGATATTCAGGTTTCCGTACATACCGAC  2100

Ye9N   2101  AGTTTGAACGAATGTGGTTATGTAGAAGACACCATTGATGCCTTCGAAGGCCGCACCATC  2160
             ||||||||||||||||||||||||||||||||||||||||||||||||||||||||||||
8081   2101  AGTTTGAACGAATGTGGTTATGTAGAAGACACCATTGATGCCTTCGAAGGCCGCACCATC  2160
```

```
Ye9N   2161  CACACTTTCCACACCGAGGGTGCGGGCGGGGGCCATGCGCCGGATATCATCCGTGTTGCC  2220
             |||||||||||||||||||| ||||||||||| |||||||||||||||||||||||||||
8081   2161  CACACTTTCCACACCGAGGGGGCGGGCGGGGGGCATGCGCCGGATATCATCCGTGTTGCC  2220

Ye9N   2221  AGTCAGCCTAACGTACTACCAAGTTCGACTAACCCAACCCTGCCATACGGGGTGAACAGC  2280
             ||||||||||||||||||||||||||||||||||||||||||||||||||||||||||||
8081   2221  AGTCAGCCTAACGTACTACCAAGTTCGACTAACCCAACCCTGCCATACGGGGTGAACAGC  2280

Ye9N   2281  CAAGCCGAACTGTTCGACATGATCATGGTGTGTCATAACCTCAACCCAAATGTACCTGCT  2340
             ||||||||||||||||||||||||||||||||||||||||||||||| ||||||||||||
8081   2281  CAAGCCGAACTGTTCGACATGATCATGGTGTGTCATAACCTCAACCCGAATGTACCTGCT  2340

Ye9N   2341  GACGTCTCCTTTGCCGAAAGCCGTGTGCGCCCGGAAACCATCGCGGCAGAAAACGTTCTG  2400
             |||||||||||||| |||||||||||||||||||||||||||||||||||||||||||||
8081   2341  GACGTCTCCTTTGCTGAAAGCCGTGTGCGCCCGGAAACCATCGCGGCAGAAAACGTTCTG  2400

Ye9N   2401  CACGATATGGGGGTTATCTCCATGTTCTCCAGTGACTCACAAGCCATGGGGCGTGTGGGG  2460
             ||||||||||||||||||||||||||||||||||||||||||||||||||||||||||||
8081   2401  CACGATATGGGGGTTATCTCCATGTTCTCCAGTGACTCACAAGCCATGGGGCGTGTGGGG  2460

Ye9N   2461  GAAAACTGGCTACGTGTGATGCAAACGGCTAATGCAATGAAAGCATCACGCGGCAAATTG  2520
             |||||||||||||||||||||||||||||||| |||||||||||||||||||||||||||
8081   2461  GAAAACTGGCTACGTGTGATGCAAACGGCTAACGCAATGAAAGCATCACGCGGCAAATTG  2520

Ye9N   2521  CCAGAAGATGCGCCGGGTAACGATAACTTCCGCGTCCTGCGCTATGTGGCAAAAATCACC  2580
             ||||||||||||||||||||||||||||||||||||||||||||||||||||||||||||
8081   2521  CCAGAAGATGCGCCGGGTAACGATAACTTCCGCGTCCTGCGCTATGTGGCAAAAATCACC  2580

Ye9N   2581  ATTAACCCAGCGATTGCACAAGGTGTCAGCCATGTCATCGGTTCAGTTGAAGTGGGCAAA  2640
             ||||||||||||||||||||||||||||||||||||||||||||||||||||||||||||
8081   2581  ATTAACCCAGCGATTGCACAAGGTGTCAGCCATGTCATCGGTTCAGTTGAAGTGGGCAAA  2640

Ye9N   2641  ATGGCCGATCTGGTGCTGTGGGATCCGCGTTTCTTTGGTGCGAAACCTAAGATGGTTATC  2700
             ||||||||||||||||||||||||||||||||||||||||||||||||||||||||||||
8081   2641  ATGGCCGATCTGGTGCTGTGGGATCCGCGTTTCTTTGGTGCGAAACCTAAGATGGTTATC  2700

Ye9N   2701  AAAGGCGGCATGATCAACTGGGCGGCAATGGGTGATCCGAACGCCTCATTACCAACCCCA  2760
             ||||||||||||||||||||||||||||||||||||||||||||||||||||||||||||
8081   2701  AAAGGCGGCATGATCAACTGGGCGGCAATGGGTGATCCGAACGCCTCATTACCAACCCCA  2760

Ye9N   2761  CAACCGGTGTTCTATCGTCCAATGTTTGGCGCAATGGGTAAAACCATGCAAGACACCTGC  2820
             ||||||||||||||||||||||||||||||||||||||||||||||||||||||||||||
8081   2761  CAACCGGTGTTCTATCGTCCAATGTTTGGCGCAATGGGTAAAACCATGCAAGACACCTGC  2820

Ye9N   2821  GTCACCTTCGTTTCTCAGGCCGCGCTGGATGATGGCGTGAAAGAGAAAGCCGGGCTGGAT  2880
             ||||||||||||||||||||||||||||||||||||||||||||||||||||||||||||
8081   2821  GTCACCTTCGTTTCTCAGGCCGCGCTGGATGATGGCGTGAAAGAGAAAGCCGGGCTGGAT  2880

Ye9N   2881  CGCCAGGTTATTGCGGTTAAAAACTGCCGTACCATCTCTAAACATGACCTGGTGCGCAAT  2940
             |||||||||||||||||||||||||||||||||||||||||||||||| ||||||| |||
8081   2881  CGCCAGGTTATTGCGGTTAAAAACTGCCGTACCATCTCTAAACATGACTTGGTGCGTAAT  2940

Ye9N   2941  GACCAAACACCAAACATTGAAGTGGATCCTGAAACCTTTGCGGTGAAAGTGGATGGCGTA  3000
             ||||||||||||||||||||||||||||||||||||||||||||||||||||||||||||
8081   2941  GACCAAACACCAAACATTGAAGTGGATCCTGAAACCTTTGCGGTGAAAGTGGATGGCGTA  3000
                                                                         codon stop
                                                                         -35
Ye9N   3001  CATGCCACCTGTGAACCTATCGATACAGCAGCAATGAACCAGCGCTATTTCTTTGGTTGA  3060
             |||||||||||||||||||| |||||||||||||||||||||||||||||||||||||||
8081   3001  CATGCCACCTGTGAACCTATAGATACAGCAGCAATGAACCAGCGCTATTTCTTTGGTTGA  3060
                                              -10
Ye9N   3061  GAGATGAGTCTGGACGTAAATCTAGTTTGTTTAAGCATTCAGTTTGCTCATGGTAAGTGT  3120
              |||||||||||||||||||||||||||||||||||||||||||||||||||||||||||
8081   3061  TAGATGAGTCTGGACGTAAATCTAGTTTGTTTAAGCATTCAGTTTGCTCATGGTAAGTGT  3120

Ye9N   3121  CATCTGGCGGTTGGATGGGGCGACCAACAATGGTCGGGATGCGTGGCATAGAAACGGATT  3180
             |||||| |||||||||||||||||||||||||||||||||||||||||||||||||||||
8081   3121  CATCTGACGGTTGGATGGGGCGACCAACAATGGTCGGGATGCGTGGCATAGAAACGGATT  3180
                                                                     >ureE
                                                                     (locus_tag=G5S39_RS14455)
Ye9N   3181  TCATTACTGTGGGCAACTGGCTTTTGCCAAAAATACAGGCAAGGAGTCTATACATGATTT  3240
             ||||||||||||||||||||||||||||||||||||||||||||||||||||||||||||
8081   3181  TCATTACTGTGGGCAACTGGCTTTTGCCAAAAATACAGGCAAGGAGTCTATACATGATTT  3240

Ye9N   3241  TGATAGAGCACATTCTTGGCAATGTGAAAAAAGATCCGGTTTGGCAGGAGAAACTCAAAG  3300
             ||||||||||||||||||||||||||||||||||||||||||||||||||||||||||||
```

```
8081   3241   TGATAGAGCACATTCTTGGCAATGTGAAAAAAGATCCGGTTTGGCAGGAGAAACTCAAAG  3300

Ye9N   3301   ATGCCACTTTTGATCTCTTGGTTTTGGATCAACGGGAAGCACAAAAAAGCCGTTGTCGTA  3360
              ||||||||||||||||||||||||||||||||||||||||||||||||||||||| ||||
8081   3301   ATGCCACTTTTGATCTCTTGGTTTTGGATCAACGGGAAGCACAAAAAAGCCGTTGCCGTA  3360

Ye9N   3361   AACTCAGCACGCAGGGGTTGGATCTGGGTATTTCGCTCGACCGACACGTCGTTCTGGCTG  3420
              ||||||||||||||||||||||||||||||||||||||||||||||||||||||||||||
8081   3361   AACTCAGCACGCAGGGGTTGGATCTGGGTATTTCGCTCGACCGACACGTCGTTCTGGCTG  3420

Ye9N   3421   ATGGGGATGTGCTGGCGTGGGATGAAAAAACCAATGTCGCGGTGGTAGTACAAATCAATT  3480
              ||||||||||||||||||||||||||||||||||||||||||||||||||||||||||||
8081   3421   ATGGGGATGTGCTGGCGTGGGATGAAAAAACCAATGTCGCGGTGGTAGTACAAATCAATT  3480

Ye9N   3481   TGCGCGATGTCATGGTTATCGATCTGAGTGAACTGAAAAGCCGTTCACCGGATGAATTGA  3540
              ||||||||||||||||||||||||||||||||||||||||||||||||||||||||||||
8081   3481   TGCGCGATGTCATGGTTATCGATCTGAGTGAACTGAAAAGCCGTTCACCGGATGAATTGA  3540

Ye9N   3541   TTAAAACCTGCTTTGAGTTGGGGCATGCACTGGGCAATCAGCACTGGAAAGCAGTGACGA  3600
              ||||||||||||||||||||||||||||||||||||||||||||||||||||||||||||
8081   3541   TTAAAACCTGCTTTGAGTTGGGGCATGCACTGGGCAATCAGCACTGGAAAGCAGTGACGA  3600

Ye9N   3601   AAAATAACGAGGTCTATGTGCCTCTGACGGTTGCCACCACCATGATGGACTCCGTGATGA  3660
              ||||||||||||||||||||||||||||||||||||||||||||||||| ||||||||||
8081   3601   AAAATAACGAGGTCTATGTGCCTCTGACGGTTGCCACCACCATGATGGATTCCGTGATGA  3660

Ye9N   3661   GAACCCACGGCTTCCAGCATTTACCTTTCCGTTTTGTTAAAGGGGCGGAAATTCTACCGT  3720
              ||||||||||||||||||||||||||||||||||||||||||||||||||||||||||||
8081   3661   GAACCCACGGCTTCCAGCATTTACCTTTCCGTTTTGTTAAAGGGGCGGAAATTCTACCGT  3720

Ye9N   3721   TACTCAGTAATTCTGAAGCACGCCTGCTGTTTGGCGGAGCTGAAGATACCGATACTCATG  3780
              ||||||||||||||||||| ||||||||||||||||||||||||||||||||||||||||
8081   3721   TACTCAGTAATTCTGAAGCGCGCCTGCTGTTTGGCGGAGCTGAAGATACCGATACTCATG  3780

Ye9N   3781   TGCATGTCGCCAGCCCTTTGGATGAACCTCATGGCTCCGGCTTACATGTTCACGCGATTC  3840
              ||||||||||||||||||||||||||||||||||||||||||||||||||||||||||||
8081   3781   TGCATGTCGCCAGCCCTTTGGATGAACCTCATGGCTCCGGCTTACATGTTCACGCGATTC  3840

Ye9N   3841   ATTCCCACGGTACTGGACATACGCATAGCCATGACCATGACCAC------------GGCG  3888
              ||||||||||||||||||||||||||||||||||||||||||||            ||||
8081   3841   ATTCCCACGGTACTGGACATACGCATAGCCATGACCATGACCACAGTCATAGCCACGGCG  3900
                                      codon stop            &gt;ureF
                                                             (locus_tag=G5S39_RS14460)
Ye9N   3889   ATCACGATCACGACCATAAACACTGATTCTGGCAGGGAGGCACCGCAATGAATGCATCAG  3948
              ||||||| ||||||||||||||||||||||||||||||||||||||||||||||||||||
8081   3901   ATCACGACCACGACCATAAACACTGATTCTGGCAGGGAGGCACCGCAATGAATGCATCAG  3960

Ye9N   3949   ATCTGATTCGTATCATGCAATTTGGTGATTCCGTACTGCCGGTCGGGGCTTTCACGTTTT  4008
              ||||||||||||||||||||||||||||||||||||||||||||||||| ||||||||||
8081   3961   ATCTGATTCGTATCATGCAATTTGGTGATTCCGTACTGCCGGTCGGGGCCTTCACGTTTT  4020

Ye9N   4009   CCAATGGCGTGGAGTCCGCCATTCAAACTGGTGTGGTACGAGACGTGCCGACGTTAAAAG  4068
              ||||||||||||||||||||||||||||||| ||||||||||||||||||||||||||||
8081   4021   CCAATGGCGTGGAGTCCGCCATTCAAACTGGCGTGGTACGAGACGTGCCGACGTTAAAAG  4080

Ye9N   4069   GCTTCGTGTTAACCGCCCCTAAAACAAGCGGCCAGTTGTGATGGCATGGGGGTGGTTGCTG  4128
              ||||||||||||||||||||||||||||||||||||||||||||||||||||||||||||
8081   4081   GCTTCGTGTTAACCGCCCCTAAAACAAGCGGCCAGTTGTGATGGCATGGGGGTGGTTGCTG  4140

Ye9N   4129   CCCATCGGGCTGTAGTAGCCGACGATCGTGACGGTATTATCCGTGCTGATTGGGCCGTGA  4188
              ||||||||||||| || || ||||| ||||||||||||||||||||||||||||||||||
8081   4141   CCCATCGGGCTGTGGTTGCTGACGACCGTGACGGTATTATCCGTGCTGATTGGGCCGTGA  4200

Ye9N   4189   ATAACCGCAAACTCAATGAAGAAAGCCGCCTGATGGCAACCCGAATGGGGAAAAAATTGG  4248
              ||||||||||||||||||||||||||||||||||||||||||||||||||||||||||||
8081   4201   ATAACCGCAAACTCAATGAAGAAAGCCGCCTGATGGCAACCCGAATGGGGAAAAAATTGG  4260

Ye9N   4249   CGGAGATGTCAATCCATGTGGTGGAGCATCCGCTGATCAGCTGGTGGCTGGAACAGATAA  4308
              ||||||||||||||||||||||||||||||||||||||||||||||||||||||||||||
8081   4261   CGGAGATGTCAATCCATGTGGTGGAGCATCCGCTGATCAGCTGGTGGCTGGAACAGATAA  4320

Ye9N   4309   AAAATGGCAATACCGCAGGGACTTACCCGGTCACTCAGGCGGTGGTGATGGCCGCACAGG  4368
              ||||||||||||||||||||||||||||||||||||||||||||||||||||||||||||
8081   4321   AAAATGGCAATACCGCAGGGACTTACCCGGTCACTCAGGCGGTGGTGATGGCCGCACAGG  4380

Ye9N   4369   GAATTGGGCAGCGCGAAGTGGTGGTGATGCACCAATATGGTGTGGCGATGACGATATTAA  4428
              | |||||||||||||||||||||||||||||||||||||| |||||||||||||||||||
```

```
8081   4381  GGATTGGGCAGCGCGAAGTGGTGGTGATGCACCAATATGGCGTGGCGATGACGATATTAA  4440

Ye9N   4429  GTGCGGCTATGCGCTTGATGCGCGTTACCCATTTCGACACACAGCATATCTTGTTTGAAT  4488
             ||||||| |||||||||||||||||||||||||||||||||||||||||||||||||| |
8081   4441  GTGCGGCCATGCGCTTGATGCGCGTTACCCATTTCGACACACAGCATATCTTGTTTGAGT  4500

Ye9N   4489  TAAACCACGACATCGAGAAGTTCTGCGATATTGCCGAAATTGGCGATATTGACCAGATGT  4548
             ||||||||||||||||||||||||||||||||||||||||||||||||||||||||||||
8081   4501  TAAACCACGACATCGAGAAGTTCTGCGATATTGCCGAAATTGGCGATATTGACCAGATGT  4560

Ye9N   4549  CTTCTTATGTCCCTATTGTGGATGTTTTGGCGGCGGTACATGTGAAAGCGCACGTTCGCC  4608
             ||||||||||||||||||||||||||||||||||||| ||||||||||||||||||||||
8081   4561  CTTCTTATGTCCCTATTGTGGATGTTTTGGCGGCGGTGCATGTGAAAGCGCACGTTCGCC  4620
                       codon stop
                           -35             -10
Ye9N   4609  TGTTTAGTAACTGATCGACTATTTAACTGATTGATAAAATGACTTCTGAGTGCTGGGTGA  4668
             ||||||||||||||||||||||||||||||||||||||||||||||||||||||||||||
8081   4621  TGTTTAGTAACTGATCGACTATTTAACTGATTGATAAAATGACTTCTGAGTGCTGGGTGA  4680

Ye9N   4669  GTGGGCTCGCCGAGGACAGCTTATTCGCCAACTACTTGGCTAATATCAGATAAAACTAAG  4728
             |||||||||||||||||||||||||||||||||||||||| |||||||||||||||||||
8081   4681  GTGGGCTCGCCGAGGACAGCTTATTCGCCAACTACTTGGCCAATATCAGATAAAACTAAG  4740

Ye9N   4729  AGGATTACCCCGTGAATAGCCATTCAACCGATAAACGCAAAAAGATCACCCGCATTGGTA  4788
             ||||||||||||||||||||||||||||||||||||||||||||||||||||||||||||
8081   4741  AGGATTACCCCGTGAATAGCCATTCAACCGATAAACGCAAAAAGATCACCCGCATTGGTA  4800
                        >ureG(locus_tag=G5S39_RS14465)
Ye9N   4789  TTGGTGGCCCGGTGGGTTCAGGCAAAACCGCCATTATCGAAGTGATCACCCCTATTCTGA  4848
             ||||||||||||||||||||||| ||||||||||||||||||||||||||||||||||||
8081   4801  TTGGTGGCCCGGTGGGTTCAGGTAAAACCGCCATTATCGAAGTGATCACCCCTATTCTGA  4860
                                                 codon stop
                                                >ureD (locus_tag=G5S39_RS14470)
Ye9N   4849  TCAAACGGGGTATTAAGCCTCTGATCATTACCAATGACATCGTCACCACCGAGGATGCCA  4908
             ||||||||||||||||||||||||||||||||||||||||||||||||||||||||||||
8081   4861  TCAAACGGGGTATTAAGCCTCTGATCATTACCAATGACATCGTCACCACCGAGGATGCCA  4920

Ye9N   4909  AACAGGTGAAACGTACCCTGAAAGGCATTCTGGATGAAGAGAAGATTCTCGGGGTCGAAA  4968
             |||||||||||||||||||||||||||||||||||||||||||||| |||||||||||||
8081   4921  AACAGGTGAAACGTACCCTGAAAGGCATTCTGGATGAAGAGAAGATCCTCGGGGTCGAAA  4980

Ye9N   4969  CCGGTGCTTGCCCGCATACTGCGGTGCGTGAAGACCCAAGTATGAATATTGCTGCGGTGG  5028
             ||||||||||||||||||||||||||||||||||||| ||||||||||||||||||||||
8081   4981  CCGGTGCTTGCCCGCATACTGCGGTGCGTGAAGACCCCAGTATGAATATTGCTGCGGTGG  5040

Ye9N   5029  AAGAGATGGAAGAGCGCTTCCCTGACAGCGACCTCATCATGATTGAAAGCGGTGGCGATA  5088
             |||||||||||||||||||||||||||||||||||||||||||||||||||||||||| |
8081   5041  AAGAGATGGAAGAGCGCTTCCCTGACAGCGACCTCATCATGATTGAAAGCGGTGGCGACA  5100

Ye9N   5089  ACCTGACACTGACCTTTAGCCCGGCCTTGGCCGACTTCTATATCTATGTCATCGATGTGG  5148
             ||||||||||||||||||||||||||||||| ||||||||||||||||||||||||||||
8081   5101  ACCTGACACTGACCTTTAGCCCGGCCTTGGCTGACTTCTATATCTATGTCATCGATGTGG  5160

Ye9N   5149  CGGAAGGGGAAAAAATCCCGCGTAAAAATGGCCCAGGTTTGGTTCAGGCGGACATTCTTG  5208
             ||||||||||||||||||||||||||||||||||||||||||||||||||||||||||||
8081   5161  CGGAAGGGGAAAAAATCCCGCGTAAAAATGGCCCAGGTTTGGTTCAGGCGGACATTCTTG  5220

Ye9N   5209  TCATCAACAAAATTGACCTCGCCCCTTATGTCGGTGCCAGCCTGGATGTGATGGAAAGTG  5268
             ||||||||||||||||||||||||||||||||||||||||||||||||||||||||||||
8081   5221  TCATCAACAAAATTGACCTCGCCCCTTATGTCGGTGCCAGCCTGGATGTGATGGAAAGTG  5280

Ye9N   5269  ACACCAAAGTGGTTCGTGGCGAGCGCCCTTATATTCTGACCAACTGCAAAACCGGGCAGG  5328
             ||||||||||||||||||||||||||||||||||||||||||||||||||||||||||||
8081   5281  ACACCAAAGTGGTTCGTGGCGAGCGCCCTTATATTCTGACCAACTGCAAAACCGGGCAGG  5340

Ye9N   5329  GAATTGAAGAGCTGGTGGATATGATTATGCGCGACTTCTTGTTTACCCATGTGCAGCCAC  5388
             | ||||||||| ||||||||||||||||||||||||||||||||||||||||||||||||
8081   5341  GCATTGAAGAGTTGGTGGATATGATTATGCGCGACTTCTTGTTTACCCATGTGCAGCCAC  5400

Ye9N   5389  AAGGAGAACATGCATGACATCGCAGAGCCAGAATATCGTGGAAACTCCTTCACGGGTTCG  5448
             |||||||||||||||||||||||||||||||||||||||||||||||| |||||||||||
8081   5401  AAGGAGAACATGCATGACATCGCAGAGCCAGAATATCGTGGAAACTCCGTCACGGGTTCG  5460

Ye9N   5449  CGCTCACGCATTAGGTATCAACGCGCCGGAACTCGCGCAATACCAAGATGAACCGGCGCA  5508
             ||||||||||||||||||||||||||||||||| ||||||||||||||||||||||||||
8081   5461  CGCTCACGCATTAGGTATCAACGCGCCGGAACTTGCGCAATACCAAGATGAACCGGCGCA  5520
```

```
Ye9N   5509   AATGCGTAGCGGGGCGGTAGGGAAAAGCGGCTATCTCAAACTGAGATTTGCCAAACGTGA   5568
              |||||||||||| |||||||||||||||||||||||||||||||||||||||||||||||
8081   5521   AATGCGTAGCGGAGCGGTAGGGAAAAGCGGCTATCTCAAACTGAGATTTGCCAAACGTGA   5580

Ye9N   5569   ACATCGCAGTATTTTGGCCGAAATGGAAAGACGGGTGCCCTCAATGGTGCAAAAAGCGCT   5628
              |||||||||||||||||||||||||||||||||||| |||||||||||||||||||||||
8081   5581   ACATCGCAGTATTTTGGCCGAAATGGAAAGACGGGTACCCTCAATGGTGCAAAAAGCGCT   5640

Ye9N   5629   GTACTGGGATGAAAAAATGCCCGAACTGCCGTGTGTCACCATGATCTCGACTTCAGGATG   5688
              ||||||||||||| ||||||||||||||||||||||||||||||||||||||||||||||
8081   5641   GTACTGGGATGAAGAAATGCCCGAACTGCCGTGTGTCACCATGATCTCGACTTCAGGATG   5700

Ye9N   5689   CATTTTACAAGGTGACCGTCTGGCCACTGACGTGATTGTGGAGGCGGGGGCTTGCGCCCA   5748
              ||||||||||||||||||||||||||||||||||||||||||||||||||||||||||||
8081   5701   CATTTTACAAGGTGACCGTCTGGCCACTGACGTGATTGTGGAGGCGGGGGCTTGCGCCCA   5760

Ye9N   5749   TGTCACTACGCAGTCGGCGACTAAAGTTCATATGATGAATGCCAACTACGCGTCGCAGAT   5808
              ||||||||||||||||||||||||||||||||||||||||||||||||||||||||||||
8081   5761   TGTCACTACGCAGTCGGCGACTAAAGTTCATATGATGAATGCCAACTACGCGTCGCAGAT   5820

Ye9N   5809   ACAGAATTTTACGGTAGAAGAAGGGGGCTATCTTGAATTTATGCCAGACCCACTTATTCC   5868
              ||||||||||||||||||||||||||||||||||||||||||||||||||||||||||||
8081   5821   ACAGAATTTTACGGTAGAAGAAGGGGGCTATCTTGAATTTATGCCAGACCCACTTATTCC   5880

Ye9N   5869   ACATCGTAATTCGCGTTTTATTACTGATACCACCATTAATATTCATCCTACGGCGACGGC   5928
              ||||||||||||||||||||||||||||||||||||||||||||||||||||||||||||
8081   5881   ACATCGTAATTCGCGTTTTATTACTGATACCACCATTAATATTCATCCTACGGCGACGGC   5940

Ye9N   5929   GATTTATTCGGAAGTGCTGATGTCTGGGCGTAAATATCACCATGCGGATGAACGCTTTGG   5988
              ||||||||||||||||||||||||||||||||||||||||||||||||||||||||||||
8081   5941   GATTTATTCGGAAGTGCTGATGTCTGGGCGTAAATATCACCATGCGGATGAACGCTTTGG   6000

Ye9N   5989   TTTTGATGTTTATTCCTCCCGAGTGGCGGCGCACGTTTTTTGGGGTAAAGAACAGCCAGC   6048
              |||||||||||||||||||||||||||||| |||||||||| ||||||||||||||||||
8081   6001   TTTTGATGTTTATTCCTCCCGAGTGGCGGCACACGTTTTTTTGGGTAAAGAACAGCCAGC   6060

Ye9N   6049   AGGTAAAGAACTGTTTGTTGAGAAATATGTGCTGGAACCGAAGTCAGAAAGTCTTGATGC   6108
              ||||||||||||||||||||||||||||||| ||||||||||||||||||||||||||||
8081   6061   AGGTAAAGAACTGTTTGTTGAGAAATATGTGTTGGAACCGAAGTCAGAAAGTCTTGATGC   6120

Ye9N   6109   TATTGGGGTAATGCAATCATTTGATGCGTTTGGCAATGTGATCTTATTAACCCCCAAAGA   6168
              ||||||||| |||||||||||||||||||| ||||||||||||||||||||||||||| |
8081   6121   TATTGGGGTCATGCAATCATTTGATGCGTTCGGCAATGTGATCTTATTAACCCCCAAACA   6180

Ye9N   6169   GCATCATGAGCGCATTTTGGCGCGGGTACCGGCCCATTTTGATATTAAAGGCGGTATTGC   6228
              |||||||||||||||| |||||||||||||||||||||||||||||||||||||||||||
8081   6181   GCATCATGAGCGCATTCTGGCGCGGGTACCGGCCCATTTTGATATTAAAGGCGGTATTGC   6240

Ye9N   6229   CAGTGGGGCAACGCGCTTACCGAATGATTGCGGGCTGGTGTTTAAAGCACTGGGAATTGA   6288
              ||||||||||||||||||||||||||||||||||||||||||||||||||||||||||||
8081   6241   CAGTGGGGCAACGCGCTTACCGAATGATTGCGGGCTGGTGTTTAAAGCACTGGGAATTGA   6300

Ye9N   6289   TAGTGCCGGTGTGAAGAATGAAATCCGACAGTTTTGGAAAATAGCTCGTGAGGAAATTCT   6348
              ||||||||||||||||||||||||||||||||||||||||||||||||||||||||||||
8081   6301   TAGTGCCGGTGTGAAGAATGAAATCCGACAGTTTTGGAAAATAGCTCGTGAGGAAATTCT   6360
                                                  codon stop
Ye9N   6349   CGGTGTGACATTGCCGGAAAAATTCTTGTGGCGTTAAGGTGTTGCTCAAAGGTATTGGCG   6408
              ||||||||||||||||||||||||||||||||||||||||||||||||||||||||||||
8081   6361   CGGTGTGACATTGCCGGAAAAATTCTTGTGGCGTTAAGGTGTTGCTCAAAGGTATTGGCG   6420

Ye9N   6409   TTGCAGCAAAACATCGCTGCAACAATAAATAGGTACAACGACGAGTGAGTGCAGCTAATA   6468
              |||||||||||||||||||||||||||||||||  |||||||||||||||||||||||||
8081   6421   TTGCAGCAAAACATCGCTGCAACAATAAATAGGG-CAACGACGAGTGAGTGCAGCTAATA   6479
                                              >yut (locus_tag=G5S39_RS14475)
Ye9N   6469   CAGCTGCGGCTTCAAGTTAGAAGGGTAAAATAATG   6503
              |||||||||||||||||||||||||||||||||||
8081   6480   CAGCTGCGGCTTCAAGTTAGAAGGGTAAAATAATG   6514
```

(B)

The *ureR*-like locus
Identities 1453/1480(98%)

```
              fliT (locus tag=G5S39_03515)
              codon stop
YE9N    1     TAATCTACCGCTAGCATGGTTATACAATCGTGTTTATAACTGAACTATCGCTTGCAACTA  60
              |||||||||||||||||||| ||||||| ||||||||||||||| |||||||||||||||
8081    1     TAATCTACCGCTAGCATGGTCATACAATAGTGTTTATAACTGAATTATCGCTTGCAACTA  60
                      -35                     -10
YE9N    61    CAA-TTATTAAAATAGCAATCAATTCAAATTGTTATTGTTTTTAACAAATAAATTTATTG  119
              ||| ||||||||||||||||||||||||||||||||||||||||||||||||||||||||
8081    61    CAATTTATTAAAATAGCAATCAATTCAAATTGTTATTGTTTTTAACAAATAAATTTATTG  120

YE9N    120   ATGGTAACAATTTTTCATGTCTGATTGACTATTATTCTCACGCCATAGCTTTTCGATAAA  179
              |||||||||||||| |||||||||||||||||||||||||| ||||||||||||| ||||
8081    121   ATGGTAACAATTTTCCATGTCTGATTGACTATTATTCTCACTCCATAGCTTTTCGCTAAA  180

YE9N    180   ATAGTCAGTGATGGTGAATTTTCTATTAAAATAAGTTGCTAGTCTGCCATTCAAAGCGTA  239
              |||||||||||||||||||||||||||||||||||||||||||| |||||||||||||||
8081    181   ATAGTCAGTGATGGTGAATTTTCTATTAAAATAAGTTGCTAGTCCGCCATTCAAAGCGTA  240
                                                   >ureR-like (locus_tag=G5S39_03510)
YE9N    240   AAGATATTCTCAGGCCAAAATTGTGAAATGAATATCGATGGAACGTACAATTAAATTTTG  299
              ||||||||||||||||||||||||||||||||||||||||||||||||||||||||||||
8081    241   AAGATATTCTCAGGCCAAAATTGTGAAATGAATATCGATGGAACGTACAATTAAATTTTG  300

YE9N    300   CCCCGGTGTGGGTAGCTCAGCACATGTTATTCAACATACAGAGCTACTCCTTACCTCAGT  359
              |||||||||||||||||| |||||||||||||||||||||||||||||||||||||||||
8081    301   CCCCGGTGTGGGTAGCTCGGCACATGTTATTCAACATACAGAGCTACTCCTTACCTCAGT  360

YE9N    360   CTATATTGAGCATCCTTTGCTCATCATGGTTAACCGTGGGCATAAAGTCATTCGCTGGGA  419
              ||||||||||||||||||||||||||||||||||||||||||||||||||||||||||||
8081    361   CTATATTGAGCATCCTTTGCTCATCATGGTTAACCGTGGGCATAAAGTCATTCGCTGGGA  420

YE9N    420   TAATAAAGAGTGCATCGTCCGGCCGGGTGAAATAGTCGCCGTCAGTAGTGGTCAAACAAT  479
              |||| |||||||||||||||||||||||||||||||||||||||||||||||||||||||
8081    421   TAATCAAGAGTGCATCGTCCGGCCGGGTGAAATAGTCGCCGTCAGTAGTGGTCAAACAAT  480

YE9N    480   TGACATAATTAATGGACTATCTACTGAGGGGCTATTTTTTAGCCACCAACTTCGCTGCGA  539
              ||||||||||||||||||||||||||||||| ||||||||||||||||||||||||||||
8081    481   TGACATAATTAATGGACTATCTACTGAGGGGTTATTTTTTAGCCACCAACTTCGCTGCGA  540

YE9N    540   TCCGCGTTTGATAACGACATTTGCTAATCACCCTGCCTCTGCAGGCCTTGGCGTTATACC  599
              |||||||||||||||||||||||||||||||||||| || ||||||||||||||||||||
8081    541   TCCGCGTTTGATAACGACATTTGCTAATCACCCTGCTTCCGCAGGCCTTGGCGTTATACC  600

YE9N    600   TGGGGTTATGCCGATGCGTGATTTGGCACCTGAATTTATTAATACTTTTGGTAATACATT  659
              ||||||||||||| ||||||||||||||||||||||||||||||||||||||||||||||
8081    601   TGGGGTTATGCCGGTGCGTGATTTGGCACCTGAATTTATTAATACTTTTGGTAATACATT  660

YE9N    660   TAAAGCAATATCCGATGTGGGTGATATCCCTCCTACTATTGTCAGGCACCGTATGCTTGA  719
              ||||||||||||||||||||||||||||||||||||||||||||||||||||||||||||
8081    661   TAAAGCAATATCCGATGTGGGTGATATCCCTCCTACTATTGTCAGGCACCGTATGCTTGA  720

YE9N    720   GTTATTACTTTGGTTAGCTCAGCGCGGTGTTAAATTTAAACTTAATGATGATCATTCATT  779
              ||||||||||||||||||||||||||||||||||||||||||||||||||||||||||||
8081    721   GTTATTACTTTGGTTAGCTCAGCGCGGTGTTAAATTTAAACTTAATGATGATCATTCATT  780

YE9N    780   AAGTGAAAAGGTACGACGCTGCCTGTCAATTGACCCACACAAAATATGGTCAGCCGCTGA  839
              ||||||||||||||||||||||||||||||||||||||||||||||||||||||||||||
8081    781   AAGTGAAAAGGTACGACGCTGCCTGTCAATTGACCCACACAAAATATGGTCAGCCGCTGA  840

YE9N    840   GGTTGCCGATCATATGGCAATGAGCGAAGTGGTATTGCGGCGAAAATTAGCGGCCGAAAA  899
              ||||||||||||||||||||||||||||||||||||||||||||||||||||||||||||
8081    841   GGTTGCCGATCATATGGCAATGAGCGAAGTGGTATTGCGGCGAAAATTAGCGGCCGAAAA  900

YE9N    900   TATACTATTACGTGATTTGATGATTGATGTCCGTATGACCAGTGCTTTACGTTTATTGCA  959
              |||||||||||||||||||||||||||||||||||||||||| |||||||||||||||||
8081    901   TATACTATTACGTGATTTGATGATTGATGTCCGTATGACCAGCGCTTTACGTTTATTGCA  960

YE9N    960   AGGTACTGATTGGCCAATTTCATTAATTGCCAGTCAAGTTGGTTATGAAAGCGCATCACG  1019
              |||||| |||||||||||||||||||||||||||||||||||||||||||||||||||||
8081    961   AGGTACAGATTGGCCAATTTCATTAATTGCCAGTCAAGTTGGTTATGAAAGCGCATCACG  1020
```

```
YE9N   1020  TTTCGCAGAACGCTTTCGTAAACGTTTTGGATTTGCTCCTACAGCCATACGCGGGCATCA  1079
             ||||||||||||||||||||||||||||||||||||||||||||||||||||||||||||
8081   1021  TTTCGCAGAACGCTTTCGTAAACGTTTTGGATTTGCTCCTACAGCCATACGCGGGCATCA  1080

YE9N   1080  TCGCTTGCAACCTATGGATACTGATCCCCCCCCAGTGAGCTTTCAGCGTTATTTAAACCT  1139
             |||||||||| |||||||||||||||||||||||||||||||||||||||||||||||||
8081   1081  TCGCTTGCAAGCTATGGATACTGATCCCCCCCCAGTGAGCTTTCAGCGTTATTTAAACCT  1140
                 codon stop
YE9N   1140  AATTTAGGGCCTGGATGAGTCTGTTCAGACTCTATTTTTAATCATTTTCGCCAGGATTTA  1199
             ||||||||||||||||||||||| ||||||||||||||| |||||||||| |||||||||
8081   1141  AATTTAGGGCCTGGATGAGTCTGCTCAGACTCTATTTTTGATCATTTTCGTCAGGATTTA  1200

YE9N   1200  CCTACCTTCCCGCCATACAACCCGTTGCATCCCCTCATTTTTTTGCTCATCATCATCCGG  1259
             ||||||||||||||||||||| ||||||||||||||| ||||||||||||||||||| ||
8081   1201  CCTACCTTCCCGCCATACAACTCGTTGCATCCCCTCA-TTTTTTGCTCATCATCATCTGG  1259

YE9N   1260  ACTGCTAAATACTGCTTTTAGATATTCTACACACTAAAACATTATTTTTTAGGCCACCTC  1319
              |||||||||||||||||||||||||||||||||||||||||||||||||||||||||||
8081   1260  GCTGCTAAATACTGCTTTTAGATATTCTACACACTAAAACATTATTTTTTAGGCCACCTC  1319

YE9N   1320  CAGTTTTTTCCTCCAGCAATACGACCTTAATATCGATAAAAGTCTTTATAATCATACAAA  1379
             ||||||||||||||||||||||||||||||||||||||||||||||||||||||||  ||
8081   1320  CAGTTTTTTCCTCCAGCAATACGACCTTAATATCGATAAAAGTCTTTATAATCATATGAA  1379

YE9N   1380  TAAATGTAAGCCACTGAAAGAAAGAGGAATATGTCACTTCTGCAAGGGCATCCTATGATT  1439
             ||||||||||||||||||||||||||||||||||||||||||||||||||||||||||||
8081   1380  TAAATGTAAGCCACTGAAAGAAAGAGGAATATGTCACTTCTGCAAGGGCATCCTATGATT  1439
                                                             >gene encoding metal-dependent
                                                             phosphohydrolase
                                                            (locus_tag=G5S39_03505)
YE9N   1440  AAAATTGGTTAAATAATATTCTCTCAGGATCGCAATGATG  1479
             ||||||||||||||||||| ||||||||||||||||||||
8081   1440  AAAATTGGTTAAATAATATCCTCTCAGGATCGCAATGATG  1479
```
