# Supplementary material for: Urease Expression in Pathogenic Yersinia enterocolitica Strains of Bio-Serotypes 2/O:9 and 1B/O:8 Is Differentially Regulated by the OmpR Regulator
Source: Front Microbiol. 2020 Apr 8;11:607. doi: 10.3389/fmicb.2020.00607 (PMC7156557; doi:10.3389/fmicb.2020.00607)
Supplement: TABLE S1 — Strains and plasmids used in this study. [file Table_1.pdf]

Supplementary Table S1. Strains and plasmids used in this study.

**Description**		**Reference**
***Y. enterocolitica* strains**		
Ye9N	derivative of clinical isolate Ye9 of bio-serotype 2/O:9, carrying virulence plasmid pYV, spontaneous Nal^R mutant	Brzostek et al., 2007
AR4	Ye9N derivative *ompR*::Km defective in OmpR production, Nal^R, Km^R	Brzostek et al., 2003
Ye8N	derivative of clinical isolate 8081 of bio-serotype 1B/O:8 restriction endonuclease-negative (R$^-$), methyltransferase-positive (M$^+$), carrying virulence plasmid pYVO8, Nal^R mutant	Kinder et al., 1993
KJ4	Ye8N derivative Δ*ompB*::Tp defective in OmpR and EnvZ production, Nal^R, Tp^R	This work
***E. coli* strains**		
BL21 (DE3)	F$^-$, *ompT*, *hsdSB*, ($r_B{}^- m_B{}^-$), *gal*, *dcm* (DE3)	Novagen
DH5α	F$^-$, *endA1*, *glnV44*, *thi-1*, *recA1*, *relA1*, *gyrA96*, *deoR*, *nupG*, *purB20*, φ80*lacZ*ΔM15, Δ(*lacZYA-argF*)U169, *hsdR17*($r_K{}^-$ $m_K{}^+$), λ$^-$	Sambrook et al., 1989
CC118 λ*pir*	*Δ(ara-leu) araD ΔlacX74 galE galK phoA20 thi-1 rpsE rpoB argE (Amr) recA1 λpir*	Herrero et al., 1990
S17 λ*pir*.	*phoA20 thi-1 rspE rpoB argE(Am) recA1 λ pir*	Simon et al., 1983
Plasmids		
pBBR1MCS-2	broad-host-range cloning vector, *ori* pBBR1, Mob$^+$, *oriT* RK2, Km^R	Kovach et al., 1995
pompB	pBBR1MCS-2 derivative carrying *ompB* cloned into BamHI and EcoRI sites	This work
p34E-Tp	source of trimethoprim cassette, Ap^R, Tp^R	Deshazer and Woods, 1996
pDS132	*ori* R6K (narrow host range, replication only in *E. coli* λpir), *oriT* RK2, *sacB*, Cm^R	Philippe et al., 2004
pDSompB	pDS132 derivative carrying 1742-bp cassette for *ompB* mutagenesis constructed by overlap extension PCR cloned between PaeI (SphI) sites of the vector, Cm^R, Tp^R	This work
pCM132Gm	pCM132 derivative, *ori* pMB1, *ori* RK2, *oriT* RK2, promoterless *lacZ* gene, Gm^R	DBG collection*
pCM132Gm-*ureABC::lacZ*	pCM132Gm derivative carrying 851 bp upstream of *ureA* start codon and 7 bp of ORF *ureA* cloned upstream of promoterless *lacZ* gene between KpnI site	This work
pCM132Gm-*ureEF::lacZ*	pCM132Gm derivative carrying 470 bp upstream of *ureE* start codon and 31 bp of ORF *ureE* cloned upstream of promoterless *lacZ* gene between EcoRI and KpnI sites	This work
pCM132Gm-*ureGD::lacZ*	pCM132Gm derivative carrying 412 bp upstream of *ureG* start codon and 33 bp of ORF *ureG* cloned upstream of promoterless *lacZ* gene between EcoRI and KpnI sites	This work
pCM132Gm-*ureR::lacZ*	pCM132Gm derivative carrying 542 bp upstream of *ureR* start codon and 52 bp of ORF *ureR* cloned upstream of promoterless *lacZ* gene between EcoRI and KpnI sites	This work
pETOmpR	pET28a carrying the entire *ompR* coding sequence (725-bp fragment), Km^R	Nieckarz et al., 2016
pBR3 (pompR)	pBBR1MCS-3 with XhoI/PstI fragment containing entire coding sequence of *ompR* (ORF with rbs), Tet^R	Brzostek et al., 2007
pRK2013	helper plasmid used to mobilize vectors in triparental mating, Km^R	Ditta et al., 1980

*Department of Bacterial Genetics, Institute of Microbiology, Faculty of Biology, University of Warsaw

Ap^R ampicillin resistance, Cm^R chloramphenicol resistance, Gm^R gentamicin resistance, Km^R kanamycin resistance, Nal^R nalidixic acid resistance, Tet^R tetracycline resistance, Tp^R trimethoprim resistance

REFERENCES

Ditta, G., Stanfield, S., Corbin, D., Helinski, D.R. (1980). Broad host range DNA cloning system for gram-negative bacteria: construction of a gene bank of *Rhizobium meliloti*. Proc. Natl. Acad. Sci. U.S.A. 77, 7347-7351.

Brzostek, K., Raczkowska, A., Zasada, A. (2003). The osmotic regulator OmpR is involved in the response of *Yersinia enterocolitica* O:9 to environmental stresses and survival within macrophages. FEMS Microbiol. Lett. 228, 265-271.

Brzostek, K., Brzóstkowska, M., Bukowska, I., Karwicka, E., Raczkowska, A. (2007). OmpR negatively regulates expression of invasin in *Yersinia enterocolitica.* Microbiol. 153, 2416-2425. doi: 10.1099/mic.0.2006/003202-0.

Deshazer, D., and Woods, D.E. (1996). Broad-host-range cloning and cassette vectors based on the R388 trimethoprim resistance gene. Biotechniques 20, 762-764.

Herrero, M., De Lorenzo, V., Timmis, K.N. (1990). Transposon vectors containing non-antibiotic resistance selection markers for cloning and stable chromosomal insertion of foreign genes in gram-negative bacteria. J. Bacteriol. 172, 6557-6567.

Kinder, S.A., Badger, J.L., Bryant, G.O., Pepe, J.C., Miller, V.L. (1993) Cloning of the YenI restriction endonuclease and methyltransferase from *Yersinia enterocolitica* serotype O8 and construction of a transformable R-M+ mutant. Gene, 1993. 136, 271-275.

Kovach, M.E., Elzer, P.H., Hill, D.S., Robertson, G.T., Farris, M.A., Roop, R.M., et al. (1995). Four new derivatives of the broad-host-range cloning vector pBBR1MCS, carrying different antibiotic-resistance cassettes. Gene. 166, 175-176.

Nieckarz, M., Raczkowska, A., Dębski, J., Kistowski, M., Dadlez, M., Heesemann, J., et al. (2016). Impact of OmpR on the membrane proteome of *Yersinia enterocolitica* in different environments: repression of major adhesin YadA and heme receptor HemR. Environ. Microbiol. 18, 997-1021, doi: 10.1111/1462-2920.13165.

Philippe, N., Alcaraz, J.P., Coursange, E., Geiselmann, J., Schneider. D. (2004). Improvement of pCVD442, a suicide plasmid for gene allele exchange in bacteria. Plasmid. 51, 246-255. doi: 10.1016/j.plasmid.2004.02.003.

Sambrook, J., Fritsch, E.F., Maniatis, T. (1989). Molecular Cloning: a Laboratory Manual. 2nd ed. Cold Spring Habor, NY, USA: Cold Spring Harbor Laboratory Press.

Simon, R., Priefer, U., Pühler, A. (1983). A broad host range mobilization system for *in vivo* genetic engineering: transposon mutagenesis in gram negative bacteria. Biotechnology. 1, 784-791.
