# Supplementary material for: Urease Expression in Pathogenic Yersinia enterocolitica Strains of Bio-Serotypes 2/O:9 and 1B/O:8 Is Differentially Regulated by the OmpR Regulator
Source: Front Microbiol. 2020 Apr 8;11:607. doi: 10.3389/fmicb.2020.00607 (PMC7156557; doi:10.3389/fmicb.2020.00607)
Supplement: TABLE S2 — Oligonucleotide primers used in this study. The table shows the sequences of primers used for cloning, the construction of transcriptional fusions, and to generate regulatory region fragments for EMSAs. [file Table_2.pdf]

Supplementary Table S2. Oligonucleotide primers used in this study. The table shows the sequences of primers used for cloning, the construction of transcriptional fusions, and to generate fragments for protein-DNA binding assays (EMSAs).

Name of primer	Primer sequence (5'→ 3')* / Restriction enzyme	Reference
**Construction of KJ4 strain**		
OmpB1m	**GCTTTGCATGC**TATCTGAAGCAGCGGCTATGG / PaeI	This study
OmpB2m	TGTCAACTGGGTTCGTGAATTCCATTTTATTACTCCCAAAGGCCGTA	This study
OmpB3m	TACGGCCTTTGGGAGTAATAAAATGGAATTCACGAACCCAGTTGACA	This study
OmpB4m	GACCACCATATTAAATAACCAAAATAAAACTCAGAATTCTTAGGCCACACGT	This study
OmpB5m	TTGAACGTGTGGCCTAAGAATTCTGAGTTTTATTTTGGTTATTTAATATGGTGGTC	This study
OmpB6m	**GCTTTGCATGC**CGCTTGCGCGAAATATTACTGCTATT / PaeI	This study
OmpB0m	CCATTGGATAATGGGGAAATC	This study
OmpB7m	TGCGTGATGGGAGTAACAGT	This study
**Construction of plasmid pompB for complementation**		
OmpB1	**TGGAATTC**CAATACGGCCTTTGGGAGTA / EcoRI	Nieckarz et al., 2016
OmpB2	**TGGGATCC**CGGCATTCACCACCATATTT / BamHI	Nieckarz et al., 2016
**Construction of *ureABC::lacZ* transcriptional fusion**		
NureABCKpnIL	**TAGGTACC**GGTGAGTGCTCTTGTGGTTTG / KpnI	This study
NureABCKpnIP	**TAGGTACC**GCTGCATAAGCCCTCCTGTA / KpnI	This study
**Construction of *ureEF::lacZ* transcriptional fusion**		
LureEFEcoRI	**TAGAATTC**CCGGTGTTCTATCGTCCAAT / EcoRI	This study
PureEFKpnI	**TAAGGTACC**CATTGCCAAGAATGTGCTCT / KpnI	This study
**Construction of *ureGD::lacZ* transcriptional fusion**		
LureGDEcoRI	**TAGAATTC**GGTGGTGATGCACCAATATG / EcoRI	This study
PureGDKpnI	**TAAGGTACC**CACTTCGATAATGGCGGTTT / KpnI	This study
**Construction of *ureR-like::lacZ* transcriptional fusion**		
EcoRIReg1	**TAGAATTC**GCTGGTTGATCTGGAAATGAC / EcoRI	This study
KpnIReg2	**TAAGGTACC**TAACATGTGCCGAGCTACCC / KpnI	This study
**Confirmation the correctness of fusions constructed in pCM132Gm**		
pCM132GmSPR1	CTGCAAGGCGATTAAGTTGG	Nieckarz et al., 2017
pCM132GmSPR2	CATAAACTGCCAGGCATCAA	Nieckarz et al., 2017
lacZSprP	GTGCTGCAAGGCGATTAAGT	This study
**EMSA *ureABC***		
ABCemL	AGTTATTTGCGATGGGAAGG	This study
ABCemP	GCTGCATAAGCCCTCCTGTA	This study
**EMSA *ureEF***		
EFemL	GCCAGGTTATTGCGGTTAAA	This study
EFemP	CACATTGCCAAGAATGTGCT	This study
**EMSA *ureGD***		
GDemL	TTGGCGATATTGACCAGATG	This study
GDemP	CACTTCGATAATGGCGGTTT	This study
**EMSA *ureR-like***		
RemL	CCCTGACCATGCTTTGCTAT	This study
RemP	TAACATGTGCCGAGCTACCC	This study
**EMSA, fragment of 16S rDNA used as a negative control**		
16SF	TACGCATTTCACCGCTAC	Jaworska et al., 2018
16SR	CAGAAGAAGCACCGGCT	Jaworska et al., 2018

*- 5' extensions added to introduce cleavage sites for the indicated restriction enzymes or epitope tag **are shown in bold.**

REFERENCES

Jaworska, K., Nieckarz, M., Ludwiczak, M., Raczkowska, A., Brzostek, K. (2018). OmpR-mediated transcriptional regulation and function of two heme receptor proteins of *Yersinia enterocolitica* bio-serotype 2/O:9. Front. Cell. Inf. Microbiol. 8: 333. doi: 10.3389/fcimb.2018.00333.

Nieckarz, M., Raczkowska, A., Dębski, J., Kistowski, M., Dadlez, M., Heesemann, J., et al. (2016). Impact of OmpR on the membrane proteome of *Yersinia enterocolitica* in different environments: repression of major adhesin YadA and heme receptor HemR. Environ. Microbiol. 18, 997-1021, doi: 10.1111/1462-2920.13165.

Nieckarz, M., Raczkowska, A., Jaworska, K., Stefańska, E., Skorek, K., Stosio D., Brzostek, K. (2017). The role of OmpR in the expression of genes of the KdgR regulon involved in the uptake and depolymerization of oligogalacturonides in *Yersinia enterocolitica*. Front. Cell. Inf. Microbiol. 7: 366. doi: 10.3389/fcimb.2017.00366.
